# The Factorial Validity of the Norwegian Version of the Multicomponent Training Distress Scale (MTDS-N)

**DOI:** 10.3390/ijerph17207603

**Published:** 2020-10-19

**Authors:** Cathrine Nyhus Hagum, Shaher A. I. Shalfawi

**Affiliations:** Department of Education and Sports Science, University of Stavanger, 4036 Stavanger, Norway; shaher.shalfawi@uis.no

**Keywords:** confirmatory factor analysis, multiple indicators multiple causes, differential item functioning, athlete monitoring, student-athletes

## Abstract

Background: Athlete self-report measures (ASRM) are methods of athlete monitoring, which have gained considerable popularity in recent years. The Multicomponent Training Distress Scale (MTDS), consisting of 22 items, is a promising self-report measure to assess training distress among athletes. The present study aimed to investigate the factorial validity of the Norwegian version of MTDS (MTDS-N) among student-athletes (*n* = 632) attending the optional program subject “Top-Level Sports” in upper secondary schools in Norway. Methods: A confirmatory factor analysis (CFA) was conducted to assess the six-factor model proposed by Main and Grove (2009). McDonald’s omega (ω) along with confidence intervals (CIs) were used to estimate scale reliability. After examining the fit of the CFA model in the total sample, covariates were included to investigate group differences in latent variables of MTDS-N, resulting in the multiple indicators multiple causes (MIMIC) model. Further, direct paths between the covariates and the factor indicators were included in an extended MIMIC model to investigate whether responses to items differed between groups, resulting in differential item functioning (DIF). Results: When modification indices (MIs) were taken into consideration, the alternative CFA model revealed that MTDS-N is an acceptable psychometric tool with a good fit index. The factors in MTDS-N all constituted high scale reliability with McDonald’s *ω* ranging from 0.725–0.862. The results indicated statistically significant group differences in factor scores for gender, type of sport, hours of training per week, school program, and school level. Further, results showed that DIF occurred in 13 of the MTDS-N items. However, after assessing the MIMIC model and the extended MIMIC model, the factor structure remained unchanged, and the model fit remained within acceptable values. The student-athletes’ reports of training distress were moderate. Conclusion: The MTDS-N was found to be suitable for use in a Norwegian population to assess student-athletes’ training distress in a reliable manner. The indications of group effects suggest that caution should be used if one is interested in making group comparisons when the MTDS-N is used among student-athletes in Norway until further research is conducted.

## 1. Introduction

The combination of sport and education, also referred to as “dual-career” [1] can be challenging for young athletes between the ages of 10 and 18 years old [2] as it demands the development of their full potential in both areas [3]. In addition to training and school loads, athletes typically encounter additional stress from other external sources such as social, work-related, lifestyle, and the athlete–coach relationship [4]. Consequently, there is a unique interaction between physical and psychological stresses [5]. Increased stresses can potentially lead to fatigue and increase the risk of illness and injury [6,7]. Hence, the balance between stress and recovery is a key factor for continuous high-level of performance [8]. Therefore, without a sufficient balance between training load and recovery, non-functional overreaching (NFOR) can occur [9]. At this stage, the first signs and symptoms of extended training distress such as performance decrements, psychological disturbance, and hormonal disturbances could occur and require weeks or months for the athlete to recover [9].

Periods of accumulated training load and changes in acute training load have also been reported to increase the risk of injury and illness [6]. Research showed that training and competition load resulted in temporary decrements in physical performance and significant levels of post-competition fatigue [10]. These decrements have been explained by increased muscle damage [11], reduction in the effectiveness of the immune system [12], an imbalance in anabolic and catabolic processes in the body [13], athlete mood disturbance [14], and a reduction in the neuromuscular effectiveness [15]. Besides training load, non-sport events can impose further stress on athletes, which shifts their physical and psychological well-being along a continuum that starts with homeostasis and progress through the stages of acute fatigue, functional overreaching, NFOR, overtraining syndrome, subclinical tissue damage, clinical symptoms, and time-loss injury or illness [16]. In normal circumstances, it can take up to five days to return to a balanced physical state (homeostasis) [13], and with increased training load and non-training stressors, it might take up to several weeks to recover [9,17]. The additional stress is not only evident in athletes playing sport at a high-performance level but also in athletes at the lower representative standards, where external pressure from schoolwork, relationship tensions, and pressure from parents and coaches has been reported [18]. Hence, there can be a risk of NFOR and overtraining (OT) for all young athletes. Consequently, this is not only an important issue for those adults that are involved in sport but also for coaches and teachers [18].

One of the challenges for those involved with athletes is to carefully monitor and manage the stresses and recovery to be able to optimize their performance capacity and to avoid harmful outcomes [19,20,21,22,23,24]. Athlete self-report measures (ASRMs) are methods of athlete monitoring, which have gained considerable popularity in recent years [25] and will likely continue growing in popularity as a monitoring strategy [26]. The utility of ASRMs as a monitoring tool is well supported and has been reported to be useful [10,23,24,27]. Their popularity stems from their low cost, easy to use, and the growing body of literature which have emphasized ASRMs to be sensitive to the risk of illness and injury, compared to physiological biomarkers [28]. An ASRM that has been considered to be promising in monitoring athletes [28] is the Multicomponent Training Distress Scale (MTDS) [29]. The instrument has been used in different sports, including swimming [30], rowing [31], soccer [32,33], cycling [34], alpine skiing [35], and tennis [36]. The instrument combines measures of mood disturbances, perceived stress, and symptoms of acute overtraining over a small number of items (22 questions) [29], and provides an insight into the intensity and frequency of psycho-behavioral responses [37]. Thus, the purpose of the present study was to translate MTDS into Norwegian (MTDS-N) and investigate whether the Norwegian version of the questionnaire can be considered a valid measure in detecting training distress among young athletes attending the optional program subject “Top-Level Sports” in upper secondary schools in Norway. Further, the study aimed to investigate the effect of covariates on the factor structure and model fit.

## 2. Materials and Methods

### 2.1. Sample Size Estimation

For the validity of the MTDS-N, the sample size was estimated using the point of stability approach, which is described in Kretzschmar and Gignac [38], Schönbrodt and Perugini [39], and the study of Hirschfeld, et al. [40]. The latter gave a direction to estimate the sample size needed for the Big Five Inventory and the International Personality Item Pool Big Five measure. The point of stability ensures that the deviation between the estimated sample and the population parameter is stable (small) and is expected to remain small at a stable statistical power = 80% [38,39]. To ensure that the stability is small, Schönbrodt and Perugini [39] indicated that, according to Cohen [41], the corridor of stability should not exceed a small correlation of 0.10. The study of Schönbrodt and Perugini [39] suggested that 240–250 participants would be the minimum number needed to reach the point of stability. Kretzschmar and Gignac [38] continued the work of Schönbrodt and Perugini [39] and reported that with perfect reliability (omega, ω = 1.0) of both latent factors and a population correlation of *p* = 0.20, the point-estimates of the correlation was stabilized at a sample size of 220 [38]. Since perfect reliability is almost never achieved, the authors suggested that the required sample at a population correlation of *p* = 0.20 and reliability of ω = 0.7 would be ≥490 participants [38]. Similar results have been reported by Hirschfeld, Brachel and Thielsch [40], and the recommended sample size to reach a point of stability was > 500 participants [40]. Therefore, the total number of participants that was required in this study was to be more or equal to the recommendations from similar studies (i.e., *n* ≥ 500).

### 2.2. Participants

The participants in the present study were 632 student-athletes attending the optional program subject Top-Level Sport from 23 different upper secondary schools in Norway. Seven covariates that characterize the profile of the respondents are presented in Table 1. The participants reported 35 different sports, which are shown in Table 2. This study was carried out according to the World Medical Association Declaration of Helsinki. Informed consent was obtained from all participants who agreed to take part in this study in accordance to the ethical approval from the Norwegian Social Science Data Services (NSD) (Project number 836079) and the Regional Committees for Medical and Health Research Ethics (REK) (Project number 54584).

### 2.3. Instrument

The MTDS was developed by Main and Grove [29] using three different instruments; the 10-item version of the Perceived Stress Scale (PSS) [42], the 24-item Brunel Mood State Scale (BRUMS) [43], and a checklist of 19 symptoms of acute overtraining [44]. The initial validation conducted by Main and Grove [29] concluded 22 items, addressing six factors. Four factors (depression, vigor, stress, and fatigue) are measured in terms of their frequency and scored on a five-point Likert scale ranging from “never” (0)–“very often” (4). The factor vigor is reversed scored, indicating that higher scores reflect the greater frequency of experiencing higher levels of energy. Further, two factors (physical symptoms and sleep disturbances) are measured in terms of their intensity and scored on a five-point Likert scale ranging from “not at all” (0) –“an extreme amount” (4). From a psychometric standpoint, the questionnaire exhibited a theoretically relevant relationship with a similar distinct construct, namely; the risk of burnout using the Athlete Burnout Questionnaire (ABQ) [29,45]. The results indicated that low scores on the ABQ resulted in low scores on the five negative training distress factors (depression, perceived stress, fatigue, sleep disturbances, and physical symptoms) and a high score on the positive factor (vigor). Conversely, high scores on ABQ resulted in high scores on the five negative training distress factors and a low score on the positive factor [29].

### 2.4. Procedures

#### Translation of the MTDS from English to Norwegian

**Figure 1:**
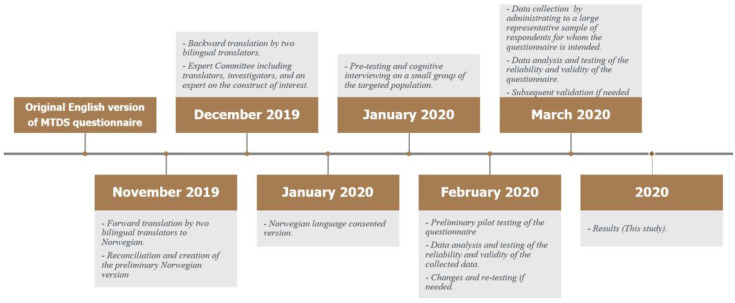
The process of translating Multicomponent Training Distress Scale (MTDS) to the Norwegian context.

Figure 1 illustrates the process of translating MTDS to the Norwegian context. The translation of the original English version to Norwegian was accomplished with reference to Guillemin, Bombardier, and Beaton [46] four-step translation procedure. Further, the International Test Commission (ITC) Guidelines for Translating and Adapting Tests were taken into consideration during the translation process [47].

In the first step, two independent bilingual, native Norwegian speakers forward translated the questionnaire from English to Norwegian. One of the translators was aware of the concepts the questionnaire intended to measure where the other was not aware of the objective of the questionnaire to offer more reliable restitution of the intended measurement [48]. A third translator compared the two versions and corrected differences to find the most appropriate words, expressions, and sentence structures to capture the meaning of the items.

In the second step, two different independent translators conducted the backward translation from Norwegian to English. To avoid bias, the translators were not familiar with the original version of the questionnaire. Both were bilingual and native English speakers. The original and backward translated versions of the questionnaire were then compared to ensure that the forward translation was precise and as complete as possible.

In the third step, an expert committee (consisting of one expert who was familiar with the construct of interest, a methodologist, one of the forward translators, and two which were not involved in the process of translations) were consulted to produce the final version of the Norwegian translation. All translated versions were reviewed with reference to achieve semantic, idiomatic, experiential, and conceptual equivalence, and any discrepancies were resolved [46].

In the fourth step, before conducting the pilot data collection of the final version of the MTDS-N, the items were tested on a small intended sample of respondents, following a probe technique [46]. Eight respondents completed the translated questionnaire and were asked verbally to elaborate on what they thought each item and their corresponding response meant. This was done in order to ensure that the final item was understood as having a meaning equivalent to that of the source item.

In the fifth step, a preliminary pilot testing of the questionnaire was carried out by distributing the questionnaire to a small group of the targeted population (*n* = 162) to measure its reliability and validity prior to the major data collection [47]. The results from the preliminary pilot testing demonstrated that the MTDS was successfully translated, culturally adapted, and reproduced the original reported psychometric properties (results of the preliminary pilot testing are attached in the Supplementary Materials). Therefore, a data collection to a larger group representing the targeted population was carried out (this study).

### 2.5. Data Collection

Invitations to participate were sent to all upper secondary schools that offer the optional program subject Top-Level Sports in Norway (*n* = 119). The final version of MTDS-N was then distributed electronically using SurveyXact version 8.0 [49] to all school management who agreed to participate in this study (*n* = 34, 28.6%). After that, the school management distributed the questionnaire electronically to the student-athletes at their respective schools (*n* = 23, 19.3%). In addition to completing the questionnaire, all participants completed questions regarding their age, gender, type of sport, hours of training per week, county, name of the school, study program, and grade level. The data collection started in March 2020 and ended in May 2020 (see Section 2.2).

### 2.6. Statistical Analysis

Prior to analyses, Microsoft Excel (version 2016) was used to prepare the data (source data are attached in the Supplementary Materials). Then, the factor vigor, with positive scores, was reversed. Demographic and descriptive data were analyzed using Statistical Package for the Social Sciences (SPSS) Version 25 (IBM Corporation, Armonk, NY, USA). Preliminary analyses investigating the normal distribution of the data were conducted using Mplus Version 8.4 (Muthén and Muthén, Los Angeles, CA, USA) [50]. The normality was examined using skewness and kurtosis (Table 3). Skewness and kurtosis values between ±1.0 were considered excellent, while values between ±1.0–2.0 were considered acceptable [51]. A non-normality test due to skewness and kurtosis was conducted to investigate if the data violated the multivariate normality assumption [52]. If the data were found not to violate the multivariate normality assumption, a Kolmogorov–Smirnov test (KS) and the Shapiro–Wilk test (SW) were further assessed to confirm that the data was normally distributed. A non-statistically significant (*p* > 0.05) Kolmogorov–Smirnov test (KS) and Shapiro–Wilk test (SW) would indicate normally distributed data [53].

All further analyses were carried out using Mplus [50]. To investigate the six-factor solution of the MTDS questionnaire proposed by Main and Grove [29], confirmatory factor analysis (CFA) was assessed. Considering a multivariate non-normality in the measures (Table 3), a maximum likelihood estimator (MLR) with robust standard errors using a numerical integration algorithm was used (Mplus codes used are attached in the Supplementary Materials).

The goodness of fit was assessed using χ^2^, root mean square error of approximation (RMSEA), comparative fit index (CFI), Tucker-Lewis index (TLI), and the standardized root mean square residual (SRMR). A good fit was indicated if the corresponding *p*-value of χ^2^ > 0.05 [54], a RMSEA value close to 0.06 [55], or a stringent upper limit of 0.07 [56], CFI and TLI ≥ 0.90 [55,57], and SRMR of ≤0.07 to indicate a good model [58], and ≤0.08 to indicate an acceptable model [55]. The model fit was further examined based on factor loadings and the estimated squared standardized factor loading (R-squared, R^2^). A factor loading of ≥0.30 was considered as the cut-off point [59,60]. To capture model misspecification, the model fit modification indices (MIs) were also taken into consideration, as CFA models with many indicators often do not fit the data [52]. High MI values would suggest freeing the corresponding parameter in the analysis if it were theoretically meaningful to do so. Together with MIs, also expected parameter change (EPC) provided information on model respecification [52]. Since the chi-square (χ^2^) statistic of the MLR cannot be used for χ^2^ difference tests, the Satorra–Bentler scaled χ^2^ difference test was used for the comparison of nested models. Further details of this procedure are given in the Mplus Web site [61]. The interpretation of effect sizes was based on the guidelines proposed by Funder and Ozer [62], where an effect size *r* of 0.05 indicated a very small effect; an effect size *r* of 0.10 indicated a small effect; an effect size *r* of 0.20 indicated a medium effect; an effect size *r* of 0.30 indicated a large effect; an effect size *r* of ≥0.40 indicated a very large effect.

A popular measure that has been widely used in social sciences to investigate internal consistency is Cronbach’s alpha (α). However, it does not provide a dependable estimate of scale reliability as it has been found to underestimate or overestimate the scale reliability depending on measurement parameters [63]. To overcome the disadvantage of Cronbach’s α, the McDonald’s omega (ω) with confidence intervals (CIs) has been recommended and applied in this study to estimate scale reliability based on the results of CFA [52,64,65,66]. The calculation of ω alongside a CI reflects the variability in the estimation process, which provides a more accurate degree of confidence in the consistency of the administration of a scale [67]. There are different reports about the acceptable values of reliability estimates, but a rule of thumb has been that it should reach 0.70 for an instrument to be acceptable [68,69]. However, very high values of α may suggest that some items are redundant as they are testing the same question but in a different way. Hence, a maximum value of reliability estimate <0.90 has been recommended [51,70] and was used as a guide in the interpretation of the ω in the preset study.

After establishing a well fitted CFA model for the total sample, covariates were included to investigate group differences in the factors from MTDS-N [71]. Such a model is referred to as multiple indicators and multiple causes (MIMIC) model [72]. The MIMIC model consists of two parts: (i) the measurement model, in which observed indicators (i.e., 22 items) measure six underlying latent factors (i.e., depression, vigor, physical symptoms, sleep disturbances, stress, and fatigue); (ii) structural equations, in which observed variables predict the six latent factors. Five covariates were included in the MIMIC model to estimate group differences on the factors, such as gender (1 = male; 2 = female), sport (1 = individual sport; 2 = team sport), hours of training per week (continuous), program (1 = specialization in general studies with Top-Level Sports; 2 = sports and physical education with Top-Level Sports), and school level (1 = first grade; 2 = second grade; 3 = third grade). Covariates labeled with the value one were considered as the reference group. Further, the MIMIC model was extended, which involved regressing the indicators and factors on the exogenous variables [73]. The purpose of the extended MIMIC model was to determine if there were any group differences in specific items, over and above differences in the latent variables [71]. Such a model is linked to differential item functioning (DIF). Differential item functioning occurs when an item has different measurement properties for one group versus another, irrespective of mean difference on the factor [74]. Detecting DIF is important since it can lead to an inaccurate conclusion about differences in groups and invalidate procedures for making decisions about individuals [75]. The factors (depression, vigor, physical symptoms, sleep disturbances, stress, and fatigue) and all endogenous indicators, except one of each latent variable, were regressed on the five covariates. This was done for the purpose of model identification [71,73]. If all direct effects between the covariates and indicators had been freely estimated at the same time, the model would be under-identified [60]. In the MIMIC models, the covariates served as grouping variables, and a significant direct effect of a covariate on a factor or item would indicate measurement non-invariance or measurement heterogeneity across the groups of the covariate (e.g., males and females).

## 3. Results

### 3.1. Item Analysis of MTDS-N

The statistical tests KS and SW yielded statistically significant (*p* < 0.001) results for all items, indicating not normally distributed data. However, in large samples, these tests can be statistically significant even when the scores are only slightly different from a normal distribution [53,76,77]. Hence, the KS and SW were interpreted in conjunction with the values of skewness (−0.02–2.09) and kurtosis (−0.08–3.97) which showed that the data were a little skewed and kurtotic. The items miserable, bitter, and depressed did not meet the criteria of ±2.0, showing kurtosis values of 3.44, 2.16, and 3.97, respectively. Furthermore, when testing for both multivariate skewness and kurtosis, the results indicate statistically significant (*p* < 0.001) results, indicating a violation of the multivariate normality assumption in the data under study.

### 3.2. Confirmatory Factor Analysis

In the first step, a CFA of the hypothesized six-factor model proposed by Main and Grove (2009) was run. The model did not fit the data well: χ^2^ = 814.824, *p*-value of χ^2^ = <0.001, RMSEA = 0.071 (90% CI: 0.066–.076), CFI = 0.873, TLI = 0.848, and SRMR = 0.057. As the hypothesized model yielded a poor fit, MIs was examined as a guide in search of model misspecification. A couple of high error covariances were specified in the model. Hence, a new alternative model was run where three error covariances (str4 with str1, MI = 147.57, EPC = 0.48; vig4 with vig3, MI = 84.13, EPC = 0.27; and fat2 with fat1, MI = 53.97, EPC = 0.33) were set as free parameters in model estimation. It appeared that the correlated items’ measurement errors in the hypothesized model were due to somewhat similar wording in the corresponding questions of the MTDS-N. After the residual covariances were set as free parameters, factor loadings were basically unchanged. Still, all the fit indices were improved with higher CFI and TLI, as well as smaller RMSEA and SRMR. The fit indices from the two CFA models are presented in Table 4.

Using the robust estimator MLR for model estimation, a scaled difference in χ^2^ was computed for nested model comparison (Table 5). The hypothesized CFA model was re-run with equality restrictions on the factor loadings to each factor, and a likelihood ratio (LR) test was conducted to test whether the indicators of each factor were equally loaded to the underlying factors. With these restrictions, the number of free parameters was reduced, the degrees of freedom of the model increased, as well as the MLR χ^2^ statistics. To compare the restricted model with the alternative model, the following formula was used for calculating the scaled difference in χ^2^ for model comparison [52]:

TRd = (T_0_ × c_0_ − T_1_ × c_1_)/c_d_

where T_0_ and T_1_ are MLR χ^2^ statistics, and c_0_ and c_1_ were the scaling correction factors for the restricted model and alternative model, respectively. For MLR, the products T_0_ ∗ c_0_ and T_1_ ∗ c_1_ were the same as the corresponding maximum likelihood (ML) χ^2^ statistics. The denominator C_d_ in the equation was the difference test scaling correction, defined as:

C_d_ = [(d_0_ × c_0_) − (d_1_ × c_1_)]/(d_0_ − d_1_)

where d_0_ and d_1_ were the degrees of freedoms for the restricted model and the alternative model. Substituting the corresponding values, the following formula was:

TR_d_ = (T_0_ × c_0_ − T_1_ × c_1_)(d_0_ − d_1_)/[(d_0_ × c_0_) − (d_1_ × c_1_)] = (1035.880 − 604.085)(204 − 191)/[204 × 1.169) − (191 × 1.155)] = 314.02(1)

Change in the model χ^2^ statistics between the restricted model and the alternative model followed a χ^2^ distribution: χ^2^ = (886.125 − 523.017) = 363.108 with the degree of freedom (df) of (204 − 191) = 13. The χ^2^ test was statistically significant (*p* < 0.001). The result indicated that restricting factor loadings equal made the model fit significantly worse than otherwise. Hence, the alternative model was preferred and retained. Standardized factor loadings and standardized R^2^ values for the two models are presented in Table 6, while inter-factor correlations from the alternative model are shown in Table 7. All factors were highly correlated (*p* < 0.001), except for the correlation between vigor and physical symptoms (*r* = 0.035, *p* = 0.535).

**Figure 2:**
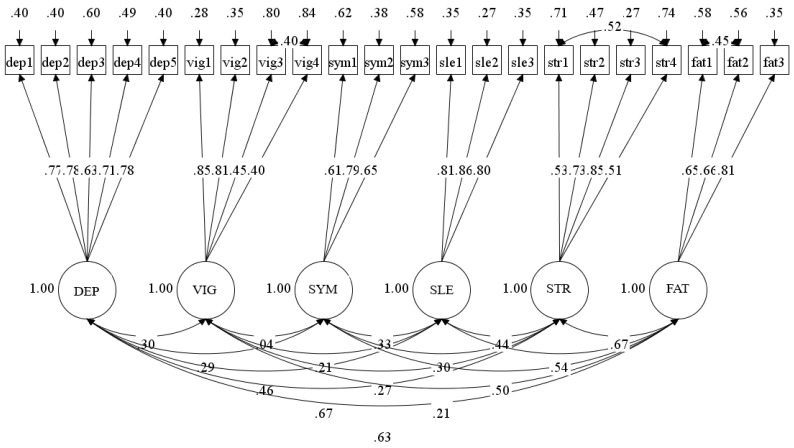
Standardized factor loadings, covariance estimates, and residual variances from the alternative model with three specified error covariances (vig3 with vig4; str1 with str4; fat1 with fat2).

As presented in Figure 2 and Table 6, standardized factor loadings ranged from 0.404–0.864, and all factor loadings were statistically significant (*p* < 0.001) and in the expected direction. The high loadings in the measurement model indicate a strong association between each of the latent factors and their respective items. The estimated R^2^ provides information about how much variance of each observed indicator variable is accounted for by its underlying factors. These values can be considered as a model estimated item reliability [52]. In the present study, sle2 has the highest R^2^ (0.732), while vig4 has the lowest (0.163).

#### Scale Reliability

The McDonald’s *ω*, along with CIs for the factors in MTDS-N, are presented in Table 8. The scale reliability estimate for depression and sleep disturbances was >0.80. The scale reliability for vigor, physical symptoms, stress, and fatigue ranged from 0.73–0.75. No estimations were above the maximum value of reliability estimate >0.90 [51,70].

To examine the extent to which athletes reported symptoms of psychophysiological stress related to training, scores from the MTDS-N were investigated. Taken collectively, as shown in Table 9, the student-athletes’ reports of training distress were moderate. Most of the factors (i.e., vigor, physical symptoms, sleep disturbances, stress, and fatigue) mean scores were between the range of “moderate amount” and “quite a bit” from the Likert-scale. The only exception was depression (M = 1.67; SD = 0.92), scoring between “a little bit” and “moderate amount.” The total score of the six factors was 13.96 (SD = 6.11).

### 3.3. Estimating Group Differences in Latent Variables

**Figure 3:**
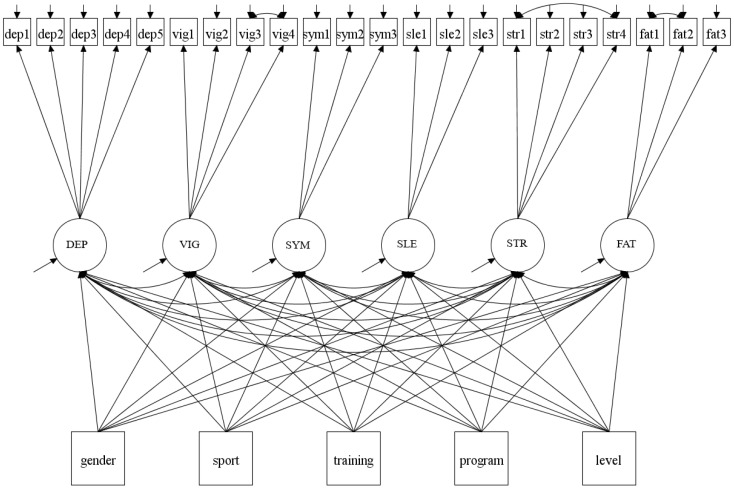
The multiple indicators multiple causes (MIMIC) model, where five covariates affect all the six factors. Gender (1 = male; 2 = female), sport (1 = individual sport; 2 = team sport), hours of training per week (continuous), program (1 = specialization in general studies; 2 = sports and physical education), and school level (1 = first grade; 2 = second grade; 3 = third grade).

In order to assess the effect of covariates on the factor structure, the MIMIC model was used. By conducting this model, the aim was to describe the relationship between the covariates and the training distress factors. Five covariates were included in the MIMIC model, such as gender (1 = male; 2 = female), type of sport (1 = individual sport; 2 = team sport), hours of training per week (continuous), school program (1 = specialization in general studies; 2 = sports and physical education), and school level (1 = first grade; 2 = second grade; 3 = third grade) were used to predict the latent variables. The same three error covariances specified in the alternative CFA model, were set as free parameters in model estimation (str4 with str1, MI = 133.12, EPC = 0.45; vig4 with vig3, MI = 94.10, EPC = 0.29; and fat2 with fat1, MI = 45.33, EPC = 0.30). Considering the multivariate non-normality in the measures, the MLR estimator was used for model estimation. Taken together, the covariates had 18 missing values (Table 1). Hence, the MIMIC model was based on a sample size of 614 participants. The model is specified in Figure 3.

After incorporating the five covariates, the factor structure remained unchanged and the model fit remained within acceptable values: χ^2^ = 808.872, *p*-value of χ^2^ < 0.001, RMSEA = 0.057 (90% CI: 0.052–0.061), CFI = 0.897, TLI = 0.871, and SRMR = 0.055. Further, the standardized (STD) results indicated that gender was a statistically significant positive predictor of the factor depression (*β* = 0.269, *p* = 0.002), physical symptoms (*β* = 0.213, *p* = 0.022), sleep disturbances (*β* = 0.448, *p* < 0.001), stress (*β* = 0.502, *p* < 0.001), and fatigue (*β* = 0.235, *p* = 0.013). The results suggest that male student-athletes tend to score lower on depression, physical symptoms, sleep disturbances, stress, and fatigue compared to female student-athletes. Participants in an individual sport tend to score lower on physical symptoms compared to team sports participants (*β* = 0.231, *p* = 0.028). Participants with fewer hours of training per week tend to score lower on physical symptoms compared to participants with more hours of training per week (*β* = 0.024, *p* = 0.020). Participants attending the school program specialization in general studies tend to score lower on depression (*β* = 0.090, *p* = 0.020), physical symptoms (*β* = 0.110, *p* = 0.007), stress (*β* = 0.105, *p* = 0.020), and fatigue (*β* = 0.094, *p* = 0.025) compared to those attending the school program sport and physical education. Contrary, participants attending the school program specialization in general studies tend to score higher on vigor (*β* = −0.237, *p* < 0.001) compared to those attending the school program sport and physical education. Furthermore, student-athletes in first grade tend to score lower on depression (*β* = 0.149, *p* = 0.008) and vigor (*β* = 0.141, *p* = 0.003), compared to student-athletes in second- and third grade. The covariates that did not have a statistically significant effect on the six training distress factors indicate invariance in the means of the factors between the groups [52]. The explained variances in the six latent variables varied from 3.1–9.4%. In detail, the covariates accounted for 4.5%, 9.4%, 3.8%, 5.9%, 8.0%, and 3.1% of the variance in the factors of depression, vigor, physical symptoms, sleep disturbances, stress, and fatigue, respectively. Table 10 presents the standardized (STD) path coefficients for the effect of the covariates on the six factors in the MIMIC model. The score values of the covariances for the different groups can be found in Supplementary Materials Table S1.

### 3.4. Estimating Group Differences in Factor Indicators

**Figure 4:**
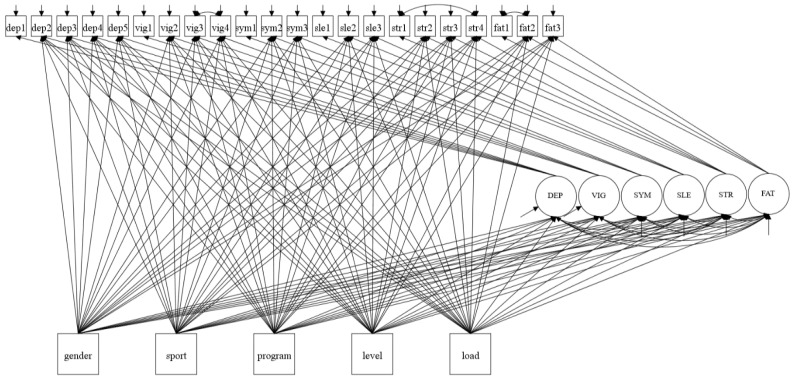
MIMIC model testing for differential item functioning (DIF). The five covariates affect all the six factors and all the items except one of each latent variable.

The MIMIC model was extended by including direct paths between the covariates and the factor indicators (i.e., MTDS-N items). The purpose of the extended model was to investigate if differences in response to items between groups would have any effect on the factor structure and the model fit. In the extended MIMIC model testing for DIF, a dummy variable was created for the covariate load (1 = more than 10 h of training per week; 0 = less than 10 h of training per week). The factors (depression, vigor, physical symptoms, sleep disturbances, stress, and fatigue) and all endogenous indicators except one of each latent variable were regressed on the covariates gender (1 = male; 2 = female), type of sport (1 = individual sport; 2 = team sport), school program (1 = specialization in general studies; 2 = sports and physical education), school level (1 = first grade; 2 = second grade; 3 = third grade), and load. To be able to identify the model, the first indicators dep1 of depression, vig1 of vigor, sym1 of physical symptoms, sle1 of sleep disturbances, str1 of stress, and fat1 of fatigue were not regressed on the covariates [52,73]. Figure 4 illustrates the extended MIMIC model testing for DIF.

After incorporating the five covariates on the extended MIMIC model testing for DIF, the factor structure remained unchanged and the model fit remained within acceptable values: χ^2^ = 414.661, *p*-value of χ^2^ *<* 0.001, RMSEA = 0.043 (90% CI: 0.038–0.049), CFI = 0.958, TLI = 0.925, and SRMR = 0.036. The results indicated that there was DIF for 13 of the items in MTDS-N. The different items with DIF are presented in Table 11.

Results indicated that gender had a statistically significant positive effect on dep2 (unhappy), dep4 (downhearted), dep5 (depressed), and sle2 (restless sleep). This result suggests that male student-athletes tend to score lower on these items compared to female student-athletes, given the same level of depression and sleep disturbances. Contrary, gender had a statistically significant negative effect on str2 (cope), str3 (piling), and fat2 (sleepy), indicating that males tend to score higher on these items compared to females, given the same level of stress and fatigue. These results imply that there are statistically significant gender differences in response to seven items, controlling for the underlying factors. However, while DIF for these items is statistically significant, it appears variously in magnitude and does not accrue systematically across the seven items. The covariate type of sport had a statistically significant positive effect on dep3 (bitter), indicating that those in an individual sport tend to score lower on the item “bitter”, compared to those in team sports, given the same level of depression. However, the magnitude of the effect was small. The covariate program had a statistically significant positive effect on vig2 (lively), vig3 (active), str2 (cope), str3 (piling), and str4 (nervous), indicating that those attending the school program specialization in general studies tend to score lower on these items compared to student-athletes attending the school program sports and physical education, controlling for the underlying factors vigor and stress. Further, the covariate program had a statistically significant negative effect on dep2 (unhappy), dep4 (downhearted), and fat3 (worn-out), indicating that those attending the school program specialization in general studies tend to score higher on these items compared to student-athletes participating the school program sports and physical education, considering the same level of depression and fatigue. The results appear variously in magnitude, from a small effect for vig3, fat3, dep2, and str4 to a very large effect for str2 and str3. Further, DIF does not accrue systematically across the eight items. The covariate level had a statistically significant negative effect on fat2 (sleepy) and fat3 (worn-out), indicating that those in first grade tend to score higher on these items compared to those in second- and third grade, controlling for the underlying factor fatigue. The effect was very small and small for the two items, respectively. Lastly, the covariate load had a statistically significant negative effect on vig3 (active) and vig4 (alert), indicating that student-athletes with less than 10 h of training per week tend to score higher on the item active and the item alert compared to student-athletes with more than 10 h of training per week, given the same level of vigor (effect was small to medium). The score values of the covariances for the different groups on the items can be found in Supplementary Materials Table S2.

## 4. Discussion

The purpose of the present study was to translate MTDS to the Norwegian context and to test the measurement instruments factorial validity, which is a form of construct validity [78]. Construct validity is essential to be able to make assumptions from scale scores about the underlying construct of interest [79]. To our knowledge, this is the first study evaluating the factor structure of MTDS by CFA. The main finding from the present study indicated that the alternative model with three error covariances set as free, fitted the data very well showing a high representativeness of all the items concerning the underlying construct of training distress. Furthermore, the MTDS-N factors scale reliability were found to be acceptable with McDonald’s *ω* ranging from 0.725–0.862. After incorporating the five covariates on the MIMIC model and the extended MIMIC model testing for DIF, the factor structure remained unchanged and the model fit remained within acceptable values. These results indicate that MTDS-N can be considered as an acceptable psychometric tool and appears to be a promising measure of training distress among Norwegian athletes.

### 4.1. Confirmatory Factor Analysis

Similar results can be observed when comparing the factor loadings from the present study with the results from Main and Grove [29]. For instance, the standardized factor loadings from the alternative model in Table 6 show a similarity in depression (0.631–0.777 vs. 0.636–0.747) and vigor (0.404–0.864 vs. 0.494–0.781). The factor alert had the lowest factor loading in both this study (0.404) and in the Main and Grove [29] study (0.494), which is in line with the low factor loading in studies where BRUMS were translated into Chinese (<0.19) [80], Malaysian (0.46) [81], and Spanish (0.16) [82]. Furthermore, factor loadings of physical symptoms (0.613–0.790 vs. −0.672–−0.790), sleep disturbances (804–0.856 vs. −0.636–−0.947), stress (0.507–0.855 vs. 0.411–0.776), and fatigue (0.650–0.806 vs. −0.502–−0.785), were also found to be quite similarly loaded. However, as shown in Table 7, the inter-factor correlations from this study were not consistent with the Main and Grove study [29]. In the Main and Grove study [29], the inter-factor correlations ranged from 0.041–0.437, with most correlations indicating medium effect sizes. In the present study, the correlations ranged from 0.035–0.668, with the most correlation indicating large to very large effect sizes. The correlations between depression and sleep disturbances (0.460), depression and stress (0.668), depression and fatigue (0.634), physical symptoms and fatigue (0.502), sleep disturbances and stress (0.441), sleep disturbances and fatigue (0.541), and stress and fatigue (0.667) were statistically significant (*p* < 0.001) and indicated very large effect sizes (Table 7). In the Main and Grove study [29], the only inter-factor correlation that yielded a very large effect size was between depression and stress (0.437). The fact that there were a few relatively high inter-factor correlations between some of the factors tells that the constructs measured can be interrelated. For example, the statistically significant (*p* < 0.001) correlation between depression and fatigue (0.634) indicates that when the value of depression increases, the value of fatigue also tends to increase. According to Puffer and McShane [83], depression and fatigue are symptoms that can be used interchangeably by athletes to describe their symptoms and feelings. Furthermore, fatigue and depression tend to be comorbid, and it has been reported that at least 30% of young people with chronic fatigue syndrome also have symptoms of depression [84]. A study by Boolani and Manierre [85] reported that depression is a predictor of long-standing feelings of fatigue in a non-athlete convenience sample [85]. Further, a statistically significant (*p* < 0.001) result was found between depression and stress (0.668). Previous studies have found statistically significant correlations between high levels of depressive symptoms and high levels of chronic stress in athletes [86,87] and women [88]. According to Brown [60], factor correlations that exceed 0.80 or 0.85 are often used as a criterion to define poor discriminant validity. In the present study, none of the correlations met this criterion; hence we can assume that the discriminant validity of the factors is good. The inter-factor correlations indicate that the domains of training distress should be regarded as factors measuring different but related aspects of training distress. This can be due to that MTDS is based on three different questionnaires, such as PSS [42], the 24-item Brunel Mood State Scale (BRUMS) [43], and a checklist of 19 symptoms of acute overtraining [44]. Nevertheless, the results from this study support the notion that the six factors can be regarded as substantially unique, as was described by Main and Grove [29], where they identified six conceptually distinct factors. In detail, the factors depression, vigor, and stress were representative of measures associated with psychological overload. The factors physical symptoms, sleep disturbances, and fatigue reflected physical and behavioral complaints associated with training distress. As such, the findings from Main and Grove [29] identified depressed mood, reduced vigor, and perceived stress as important psychological indicators of training distress. Further, their findings confirmed that physical symptoms, sleep disturbances, and general fatigue were behavioral correlates of training distress.

#### Scale Reliability

The scale reliability for the factors in MTDS-N was also acceptable with McDonald’s *ω* ranging from 0.725–0.862. To our knowledge, no other studies have used McDonald’s *ω* regarding scale reliabilities for the MTDS factors. However, other studies have reported Cronbach’s α. The internal consistency presented by Main and Grove [29] showed values of α ranging from 0.72–0.86, and the six-factor solution accounted for 67.01% of the common item variance. The following Cronbach’s α has been reported from a study on alpine skiers: depressed = 0.84, vigor = 0.76, physical symptoms = 0.50, sleep disturbances = 0.87, stress = 0.81, and fatigue = 0.80 [35]. Another study reported the overall internal consistency as α = 0.90 [89]. Other studies that have used the MTDS have not reported values of α, or any other measure of scale reliability [31,33,34,36]. Collectively, the scale from the present study constitutes high scale reliability when compared with other studies that have used the same instrument. However, it is important to keep in mind the limitations that are associated with Cronbach’s α as it has been found to underestimate or overestimate the scale reliability depending on measurement parameters [63]. Hence, it does not provide a dependable estimate of scale reliability, and for this reason, the McDonald’s ω with CIs has been recommended and applied in this study to estimate scale reliability based on the results of CFA [52,64,65,66].

### 4.2. Estimating Group Differences in Latent Variables

The MIMIC model was conducted to investigate whether factor means were different between groups and to assess the effect of covariates on the factor structure and goodness of fit. The results from the present study indicated that the estimated factor structure remained unchanged and the model fit remained within acceptable values (χ^2^ = 808.872, *p*-value of χ^2^ < 0.001, RMSEA = 0.057 (90% CI: 0.052–0.061), CFI = 0.897, TLI = 0.871, and SRMR = 0.055) after incorporating the five covariates to the model. Further, the analysis indicated statistically significant differences in factor scores for *gender* on the factors of depression, physical symptoms, sleep disturbances, stress, and fatigue. The statistically significant effect of gender on the MTDS-N factors represent population heterogeneity; that is, the factor means are different at different levels of the covariate gender [60]. Population heterogeneity in MTDS has also been reported showing that females have overall higher scores than males, indicating differing mood disturbances between the genders [32,90]. The MTDS is a recently developed ASRM instrument and hence less investigated [28]; however, similar results regarding gender differences for PSS, which include some of the same symptoms as in the MTDS, have been reported. Those results indicate that women tend to score significantly higher on PSS scores compared to men [91]. Further, a prospective study on young elite athletes revealed that females reported more stress and more depressive symptoms, compared to males [92]. Interestingly, there were no statistically significant differences in vigor factor scores for gender, indicating invariance in the factor means. Hence, the probability of a student-athlete receiving an observed score is not dependent on the individuals’ gender, but the individuals’ true score [93]. Nevertheless, research shows that females most often score consistently higher than males on instruments measuring negative characteristics [94,95,96]. The finding from the present study corresponds with previous research [94,95,96], where population heterogeneity was found for the negative symptoms and not for the positive symptoms from the factor vigor. However, it is not clear whether this trend is a result of reasonable gender differences in terms of the latent constructs being measures or caused by other secondary factors [94]. According to Terry, et al. [97], there are a number of theories and empirical attempts to explain gender disparity, among others, these differences are artifacts of measurement bias and not true differences between males and females. An artifact explanation is based on the hypothesis that males may be less willing than females to admit negative symptoms [98]. Thus, rates of the negative symptoms may be equivalent in males and females; however, depressive symptoms are perceived as less masculine, which could result in males unwillingness to report such symptoms [99,100,101]. The indication of gender differences suggests that caution should be taken if group comparison is the intended purpose when using the MTDS-N among student-athletes.

The results of the present study showed a statistically significant difference in physical symptoms factor scores for the type of sport, suggesting that participants from individual sports tend to score lower on physical symptoms compared to participants from team sports. This finding is not in line with previous research where it has been reported that athletes from individual sports are more likely to report anxiety and depression compared to team sport athletes [102,103,104], which is explained by the fact that team sports athletes, throughout adolescence, tend to have a protective effect against depressive symptoms compared to individual sport athletes [105]. Conversely, no statistically significant differences were observed for depression, vigor, sleep disturbances, stress, and fatigue (Table 10), which are in line with findings from Birrer, et al. [106], indicating no statistically significant differences in the prevalence of training distress and overtraining syndrome between individual sport and team sports. A potential explanation for this finding can be linked to differences in the practice of sport in a given country. Differences between countries exist based on the nation’s geographical, economic, social, historical, political, and cultural profile [107,108,109].

Regarding the covariate hours of training, results indicated statistically significant differences in factor scores of physical symptoms. There were no statistically significant differences in factor scores for the other factors in MTDS-N. Although the effect was small, this result suggests that participants with fewer hours of training per week tend to score lower on physical symptoms compared to participants with more hours of training per week. Previous research has indicated a clear effect of training load on soreness and neuromuscular fatigue in rugby athletes [110]. Another study revealed that muscle soreness is moderately related to the daily training load in professional soccer players [111]. Training and competition load results in temporary decrements in physical performance and significant levels of post-competition fatigue [10]. These decrements have been explained by increased muscle damage [11], reduction in the effectiveness of the immune system [12], an imbalance in anabolic and catabolic processes in the body [13], athlete mood disturbance [14], and a reduction in the neuromuscular effectiveness [15].

The covariate school program was a statistically significant positive predictor for the factors of depression, physical symptoms, stress, fatigue, and a statistically significant negative predictor of vigor. Hence, indicating that participants attending the school program specialization in general studies tend to score lower on depression, physical symptoms, stress, and fatigue compared to those attending the school program sport and physical education. Contrary, participants attending the school program specialization in general studies tend to score higher on vigor compared to those attending the school program sport and physical education. This could be explained by the fact that, in Norway, athletes attending the school program sport and physical education have more subjects involving physical training compared to students attending specialization in general studies. Further, the finding can be linked to the statistically significant result regarding the covariate hours of training, suggesting that participants with more hours of training per week tend to score higher on physical symptoms compared to participants with fewer hours of training per week.

School level was a statistically significant positive predictor for the factor depression and vigor, indicating that student-athletes in first grade tend to score lower on depression and vigor, compared to student-athletes in second- and third grade. Previous research has indicated that freshmen (first year) and sophomores (second year) have higher training distress scores compared to juniors (third year) and seniors (fourth year), and for this reason, year in school has been identified as a possible variable that could serve as an indicator of training distress [32]. A study by Gustafsson, et al. [112] that used the Profile of Mood States (POMS) [113] discussed that vigor might be an important indicator of maladaptation and NFOR. For example, fatigue is more sensitive and captures general training fatigue, whereas a decrease in vigor might indicate a more severe state. According to Meeusen, Duclos, Gleeson, Rietjens, Steinacker and Urhausen [9], when the balance between training and recovery is not sufficiently respected, symptoms of prolonged training distress, including decreased vigor, will occur, leading to NFOR. However, a possible explanation of the results of vigor in this study could be attributed to the fact that the student-athletes in the first grade are fresh comers and not adapted to the increased training load, suggesting that school coaches and club coaches should pay attention to the total training load for fresh student-athletes. Another potential explanation for decreased vigor among student athletes in first grade might be due to biological reasons. Boolani, et al. [114] found that feelings of vigor are associated with mitochondrial function, which is usually lower in people who are not as well trained and those who are younger and do not have as much muscle mass. Further, their findings suggest that vigor is associated with normalized resting metabolic rate, which is usually higher in those who are not well trained [114].

### 4.3. Estimating Group Differences in Factor Indicators

The extended MIMIC model was conducted to investigate if there existed DIF in the responses of MTDS-N by examining the effect of covariates on factor indicators (i.e., items) and to assess if DIF would have an effect on the factor structure and goodness of fit. Such analysis can be considered as an extended method of construct validity, taking variables outside the questionnaire into account [115]. The main findings indicated that the estimated factor structure remained unchanged and the model fit remained within acceptable values (χ^2^ = 414.661, *p*-value of χ^2^ < 0.001, RMSEA = 0.043 (90% CI: 0.038–0.049), CFI = 0.958, TLI = 0.925, and SRMR = 0.036). However, the results indicated that 13 of 22 items exhibited statistically significant DIF. Responses to scale items were mostly affected by gender (seven DIF) and school program (eight DIF). However, the impacts of gender and school program on item responses were not systematic across the item set (i.e., four of seven items exhibited positive DIF for gender and five of eight items exhibited positive DIF for school program). The effect of the school program on item response was notable because two of the items (str2 and str3) were very large in magnitude (*β* > 0.50). The results of DIF in the present study indicate that the MTDS-N items functions differently for different groups; that is, they have a different probability of giving a certain response to the corresponding item given the same underlying factor score [116]. However, investigating the CFA factor loadings indicates that DIFs have been canceled out at the total test score. This means that while males and females have seven DIF and participants attending the school program specialization in general studies and participants attending the school program sport and physical education have eight DIF, differences were small in magnitude and their effect on the sociability dimension were negligible (Table 11). What are the practical consequences of the DIF in MTDS-N? Whether bias matters depends not just on the amount of bias, but also the purposes of the researcher [117]. Hence, one could shift the question from “is the test biased?” to “does the amount of bias in the test matter?”. This shifting is especially vital because DIF would be detected in all items of all scales with sufficiently large samples [117]. In the present study, most of the statistically significant DIF was small in magnitude (Table 11). Borsboom [117] considers three possible uses of the test score. Firstly, if a researcher is interested in comparing means, biasing effects may be negligible if they are small in magnitude. Thus, violations of measurement invariance do not need to be a serious threat to validity. Secondly, if a researcher is interested in comparing within-group relations, bias may be entirely irrelevant. Finally, if the purpose is to select specific individuals (e.g., selection of diseases), then measurement invariance is a necessary condition for fair selection. However, further investigations are recommended to produce a more nuanced picture of the presence of DIF in the MTDS-N. If the scale is to be modified, different authors have proposed solutions to handle the presence of DIF in practice [118]. According to the authors of the review, researchers have recommended to split items exhibiting DIF to calibrate them in each group separately when the scale is used in a study; to remove items exhibiting DIF from the scale; or reformulate items exhibiting DIF [118].

The results from the present study must be considered in light of some limitations. First, data are based on self-report, which can result in response bias [20,119]. Additionally, the purpose of this study was to investigate the psychometric properties of the Norwegian version of MTDS, and therefore the data was collected at a single time point. Hence, a longitudinal approach would be ideal for investigating the perceptions captured by the MTDS-N over time. Regarding the choice of statistical analysis, the MIMIC model can only test non-invariances in factor means and item intercepts. To test non-invariance in factor loadings, factor variances, and measurement error variances, a multigroup CFA would be preferable. However, the MIMIC model has some advantages compared to the multigroup CFA. First, it does not require a large sample size. Further, it is possible to include continuous measures for the covariates in the MIMIC model, which is not appropriate for multigroup CFA [52].

## 5. Conclusions

The main objective of the present study was to examine the validity and reliability of the translated English version of MTDS into the Norwegian language to be able to assess the psychometric properties among Norwegian student-athletes. The alternative CFA model reported in this study yielded acceptable fit indices and strong scale reliability, indicating the suitability of the MTDS-N to be used in a Norwegian population to assess student-athletes training distress. There were indications of group effects, suggesting that different groups could score differently on the MTDS-N. Thus, caution is required if group comparison is the intended purpose when using the MTDS-N among student-athletes.

## Figures and Tables

**Table 1:** The profile of the 632 student-athletes in the present study.

Characteristics (Total) ^1^	Modalities	Frequency or M ± SD	%
Gender (630)	Male	327	51.9
	Female	303	48.1
Type of sport (630)	Individual	207	32.9
	Team sport	423	67.1
Region (632)	West Norway	344	54.4
	East Norway	148	23.4
	Mid Norway	160	16.8
	Northern Norway	34	5.4
Age in years (631)	Male	17.37 ± 0.06	
	Female	17.23 ± 0.05	
Training hours (617)	Total	12.54 ± 4.99	
	Specialization in general studies	12.60 ± 4.95	
	Sports and physical education	12.45 ± 5.06	
School program ^2^ (632)	Specialization in general studies	369	58.4
	Sports and physical education	263	41.6
School level ^3^ (632)	First grade	232	36.7
	Second grade	239	37.8
	Third grade	161	25.5

Notes. M = mean; SD = standard deviation; % = percentage. ^1^ Values in brackets indicate total responses from the participants. There were 20 missing values, but the number of cases with missing values on the characteristics was 18. ^2^ In the education program specialization in general studies with Top-Level Sports, the student-athletes are attending regular specialization in general studies with Top-Level sports as an optional program subject. Thus, they have only theoretical subjects in addition to the physical Top-Level sports subject. In the education program sports and physical education, the student-athletes have many subjects that are related to sports, both theoretical and practical. The subjects are activity theory, theory of training, training management, sports and society, and the optional program subject Top-Level Sports. Hence, student-athletes connected to the program sports and physical education have more hours of training per week at school, compared to those connected to the program specialization in general studies. ^3^ In Norway, the ages of the students are 15–16 years in first grade, 16–17 years in second grade, and 17–18 years in third grade. These ages can be compared to sophomores, juniors, and seniors, respectively, in high schools in the United States.

**Table 2:** The different sports reported by the 630 participants (two missing).

Descriptive Statistics					
Type of Sport	Frequency	%	Type of Sport	Frequency	%
Soccer	306	48.6	Sailing	6	1.0
Handball	91	14.4	Martial art	9	1.4
Swimming	24	3.8	Badminton	5	0.8
Track field	21	3.3	Cheerleading	1	0.2
Gymnastics	11	1.7	Strength training	4	0.6
Ice hockey	19	3.0	Sky jumping	1	0.2
Cross-country skiing	34	5.4	Diving	1	0.2
Orienteering	8	1.3	Sports drill	4	0.6
Alpine skiing	15	2.4	Shooting	1	0.2
Cycling	12	1.9	Snowboard	1	0.2
Golf	5	0.8	Jet ski	1	0.2
Floorball	2	0.3	Dance	1	0.2
Volleyball	5	0.8	Motocross	2	0.3
Rowing	3	0.5	Triathlon	2	0.3
Biathlon	12	1.9	Freeski	1	0.2
Show jumping	12	1.9	Climbing	1	0.2
Ice skate	4	0.6	Figure skating	1	0.2
Tennis	4	0.6			

**Table 3:** Descriptive statistics for 632 participants on the items of MTDS-N.

Items	Descriptive Statistics			
	M	SD	Skewness	Kurtosis
Depression (dep1–dep5)				
Miserable (dep1)	1.47	0.82	1.95	3.44
Unhappy (dep2)	1.75	0.94	1.27	1.09
Bitter (dep3)	1.64	0.86	1.49	2.16
Downhearted (dep4)	2.03	1.06	0.92	0.11
Depressed (dep5)	1.49	0.90	2.09	3.97
Vigor (vig1–vig4)				
Energetic (vig1)	2.70	0.99	0.38	−0.08
Lively (vig2)	2.61	0.95	0.54	0.03
Active (vig3)	2.52	0.90	0.32	−0.24
Alert (vig4)	2.87	0.94	0.30	−0.21
Physical symptoms (sym1–sym3)				
Muscle soreness (sym1)	2.52	1.03	0.18	−0.68
Heavy arms or legs (sym2)	2.43	0.98	0.38	−0.44
Stiff/sore joints (sym3)	2.11	1.03	0.73	−0.19
Sleep disturbances (sle1–sle3)				
Difficulties falling asleep (sle1)	2.15	1.18	0.84	−0.32
Restless sleep (sle2)	2.06	1.16	0.90	−0.21
Insomnia (sle3)	1.83	1.11	1.22	0.51
Stress (str1–str4)				
Stressed (str1)	3.06	1.11	−0.02	−0.65
Could not cope (str2)	2.76	1.02	0.10	−0.46
Difficulties piling up (str3)	2.12	0.96	0.68	0.08
Nervous (str4)	2.78	1.09	0.15	−0.56
Fatigue (fat1–fat3)				
Tired (fat1)	2.69	0.98	0.28	−0.42
Sleepy (fat2)	2.54	1.09	0.43	−0.55
Worn-out (fat3)	2.46	1.07	0.41	−0.59

Notes. M = mean; SD = standard deviation; Dep = depression; Vig = vigor; Sym = physical symptoms; Sle = sleep disturbances; Str = stress; Fat = fatigue.

**Table 4:** The test of model fit from the six-factor solution proposed by Main and Grove (2009) and the alternative model taking three measurement errors into consideration.

Fit Indices	The Hypothesized Model	The Alternative Model
χ^2^	814.824	523.017
df	194	191
*p*	<0.001	<0.001
RMSEA	0.071	0.052
CI	0.066–0.076	0.047–0.058
CFI	0.873	0.932
TLI	0.848	0.918
SRMR	0.057	0.050

Notes. χ^2^ = chi-square value; Df = degree of freedom; *p* = probability value of χ^2^; RMSEA = root mean square error of approximation; CI = confidence interval; CFI = comparative fit Index; TLI = Tucker–Lewis index; SRMR = standardized root mean square residual.

**Table 5:** Calculating the scaled difference in chi-square for nested model comparison using the robust estimator MLR.

MLR			ML	
Alternative model				
T_1_ 523.017	d_1_ 191	c_1_ 1.155	T_1_ × c_1_ 604.085	d_1_ 191
Restricted model				
T_0_ 886.125	d_0_ 204	c_0_ 1.169	T_0_ × c_0_ 1035.880	d_0_ 204

Note. MLR: robust maximum likelihood; ML: maximum likelihood; Alternative model: modified six-factor CFA of the MTDS-N; T_1_: MLR chi-square statistic for the alternative model; d_1_: the degree of freedom (df) for the alternative model; c_1_: scaling correction factor for the alternative model. Restricted model: six-factor CFA with restricted factor loadings; T_0_: MLR chi-square statistic for the restricted model; d_0_: df for the restricted model; c_0_: scaling correction factor for the restricted model.

**Table 6:** Standardized factor loadings and R^2^ values for each item in the questionnaire for the hypothesized model and the alternative model.

Item	Hypothesized	R^2^	Alternative	R^2^
Miserable (dep1)	0.768	0.590	0.773	0.598
Unhappy (dep2)	0.782	0.611	0.777	0.604
Bitter (dep3)	0.632	0.400	0.631	0.399
Downhearted (dep4)	0.715	0.512	0.713	0.508
Depressed (dep5)	0.773	0.598	0.775	0.601
Energetic (vig1)	0.830	0.689	0.864	0.716
Lively (vig2)	0.798	0.637	0.805	0.648
Active (vig3)	0.498	0.248	0.451	0.204
Alert (vig4)	0.455	0.207	0.404	0.163
Muscle soreness (sym1)	0.614	0.377	0.613	0.376
Heavy arms or legs (sym2)	0.789	0.623	0.790	0.625
Stiff/sore joints (sym3)	0.650	0.423	0.650	0.422
Difficulty falling asleep (sle1)	0.803	0.645	0.805	0.649
Restless sleep (sle2)	0.855	0.732	0.856	0.732
Insomnia (sle3)	0.806	0.649	0.804	0.646
Stressed (str1)	0.627	0.393	0.534	0.285
Could not cope (str2)	0.699	0.489	0.726	0.527
Difficulties piling up (str3)	0.809	0.654	0.855	0.731
Nervous (str4)	0.601	0.361	0.507	0.257
Tired (fat1)	0.797	0.635	0.650	0.422
Sleepy (fat2)	0.809	0.655	0.664	0.440
Worn-out (fat3)	0.700	0.490	0.806	0.649

Note. R^2^ = coefficient of determination.

**Table 7:** Standardized inter-factor correlations from the alternative model above the diagonal and inter-correlations from the initial study of MTDS are presented below the diagonal.

Factor	Depression	Vigor	Physical Symptoms	Sleep Disturbances	Stress	Fatigue
DEP	1	0.304 **	0.292 **	0.460 **	0.668 **	0.634 **
VIG	−0.194	1	0.035	0.207 **	0.269 **	0.207 **
SYM	−0.228	0.041	1	0.331 **	0.305 **	0.502 **
SLE	−0.394	0.110	0.247	1	0.441 **	0.541 **
STR	0.437	−0.259	−0.181	−0.273	1	0.667 **
FAT	−0.208	0.182	0.321	0.207	−0.311	1

Notes. ** = *p* < 0.001.

**Table 8:** Calculated McDonald’s ω along with confidence intervals (CIs) to estimate scale reliability.

Factor	Estimate	Lower 5% CI	Upper 5% CI
Depression	0.853	0.831	0.887
Vigor	0.747	0.714	0.799
Physical symptoms	0.725	0.690	0.779
Sleep disturbances	0.862	0.841	0.895
Stress	0.745	0.715	0.739
Fatigue	0.753	0.717	0.809

Note. CI = confidence interval.

**Table 9:** Mean scale scores for the six factors in MTDS-N.

Factor	Descriptive Statistics	
	M	SD
1. Depression (dep)	1.67	0.92
2. Vigor (vig)	2.67	0.94
3. Physical symptoms (sym)	2.35	1.01
4. Sleep disturbances (sle)	2.01	1.15
5. Stress (str)	2.68	1.05
6. Fatigue (fat)	2.56	1.05
Total score ^a^	13.96	6.11

^a^ Total score represents the sum of the six MTDS factors.

**Table 10:** MIMIC model results of the covariates gender, age, type of sport, hours of training per week, county, school program, and school level on the factors depression, vigor, physical symptoms, sleep disturbances, stress, and fatigue.

Factor (Explained Variances)	Covariates	*β*	S.E.	*p*
Depression (0.045 = 4.5%)	Gender	0.269	0.086	0.002 *
	Sport	−0.172	0.103	0.096
	Training	−0.008	0.010	0.445
	Program	0.090	0.038	0.020 *
	Level	0.149	0.057	0.008 *
Vigor (0.094 = 9.4%)	Gender	0.135	0.079	0.089
	Sport	−0.062	0.092	0.501
	Training	−0.011	0.007	0.143
	Program	−0.237	0.038	0.000 **
	Level	0.141	0.048	0.003
Physical symptoms (0.038 = 3.8%)	Gender	0.213	0.093	0.022 *
	Sport	0.231	0.105	0.028 *
	Training	0.024	0.010	0.020 *
	Program	0.110	0.040	0.007 *
	Level	−0.008	0.061	0.895
Sleep disturbances (0.059 = 5.9%)	Gender	0.448	0.086	0.000 **
	Sport	−0.090	0.100	0.370
	Training	−0.012	0.008	0.163
	Program	0.044	0.034	0.193
	Level	0.073	0.055	0.186
Stress (0.080 = 8.0%)	Gender	0.502	0.089	0.000 **
	Sport	−0.042	0.105	0.686
	Training	−0.012	0.009	0.207
	Program	0.105	0.045	0.020 *
	Level	0.079	0.056	0.159
Fatigue (0.031 = 3.1%)	Gender	0.235	0.094	0.012 *
	Sport	0.048	0.106	0.650
	Training	−.016	0.009	0.090
	Program	0.094	0.042	0.025 *
	Level	0.066	0.064	0.306

Notes. S.E. = standard error; *β* *=* beta; * = *p* < 0.05; ** = *p* < 0.001.

**Table 11:** Standardized (STD) model results for the MIMIC model testing DIF with the interpretation of effect sizes.

Indicators	Covariates	*β*	S.E.	*p*	Effect Size
dep2 (unhappy)	Gender	0.255	0.072	0.000 **	M
	Program	−0.194	0.045	0.000 **	S
dep3 (bitter)	Sport	0.164	0.072	0.023 *	S
dep4 (downhearted)	Gender	0.287	0.075	0.000 **	M
	Program	−0.213	0.043	0.000 **	M
dep5 (depressed)	Gender	0.182	0.064	0.004 *	S
vig2 (lively)	Program	0.231	0.046	0.000 **	M
vig3 (active)	Program	0.143	0.033	0.000 **	S
	Load	−0.174	0.069	0.012 *	S
vig4 (alert)	Load	−0.200	0.072	0.006 *	M
sle2 (restless sleep)	Gender	0.181	0.075	0.016 *	S
str2 (cope)	Gender	−0.295	0.108	0.006 *	M
	Program	0.528	0.061	0.000 **	VL
str3 (piling)	Gender	−0.369	0.111	0.001 *	L
	Program	0.559	0.062	0.000 **	VL
str4 (nervous)	Program	0.151	0.044	0.001 *	S
fat2 (sleepy)	Gender	−0.212	0.070	0.002 *	M
	Level	−0.090	0.045	0.047 *	VS
fat3 (worn-out)	Program	−0.107	0.047	0.017 *	S
	Level	−0.177	0.060	0.003 *	S

Note. * = *p* < 0.05; ** = *p* < 0.001; VS = very small; S = small; M = medium; L = large; VL = very large; sym2, sym3 and sle3 were DIF-free and were not included in the table.

## Supplementary Materials

The following are available online at https://www.mdpi.com/1660-4601/17/20/7603/s1. Table S1: Score values of on the factors for the different groups; Table S2: Score values of the factor predictors for the different groups; results of the preliminary pilot testing; results of the preliminary pilot testing with new model with “BY” statement; Mplus Code; source data.
