# Supplementary material for: The Factorial Validity of the Norwegian Version of the Multicomponent Training Distress Scale (MTDS-N)
Source: Int J Environ Res Public Health. 2020 Oct 19;17(20):7603. doi: 10.3390/ijerph17207603 (PMC7590227; doi:10.3390/ijerph17207603)
Supplement: Supplementary file 1 [file ijerph-17-07603-s001.zip › ijerph-944040-Supplementary/Supplementary Materials.docx]

**Supplementary**

| Table S1. Score values of the factors for the different groups. | | | |
| --- | --- | --- | --- |
| Factor | Characteristics | Modalities | M ± SD |
| DEP | Gender | Male | 7.90 ± 3.38 |
|  |  | Female | 8.83 ± 3.88 |
|  | Type of sport | Individual | 8.83 ± 4.23 |
|  |  | Team sport | 8.14 ± 3.40 |
|  | Training hours | < 10 hours | 8.45 ± 3.58 |
|  |  | > 10 hours | 8.33 ± 3.79 |
|  | School program | Specialization in general studies | 8.35 ± 3.59 |
|  |  | Sports and physical education | 8.41 ± 3.88 |
|  | School level | First grade | 7.96 ± 3.53 |
|  |  | Second grade | 8.59 ± 3.83 |
|  |  | Third grade | 8.66 ± 3.74 |
| VIG | Gender | Male | 10.41 ± 2.81 |
|  |  | Female | 11.01 ± 2.96 |
|  | Type of sport | Individual | 10.83 ± 3.09 |
|  |  | Team sport | 10.63 ± 2.80 |
|  | Training hours | < 10 hours | 11.00 ± 2.72 |
|  |  | > 10 hours | 10.50 ± 2.98 |
|  | School program | Specialization in general studies | 10.77 ± 2.93 |
|  |  | Sports and physical education | 10.60 ± 2.83 |
|  | School level | First grade | 10.39 ± 2.96 |
|  |  | Second grade | 10.86 ± 3.03 |
|  |  | Third grade | 10.89 ± 2.53 |
| SYM | Gender | Male | 6.88 ± 2.47 |
|  |  | Female | 7.23 ± 2.42 |
|  | Type of sport | Individual | 6.97 ± 2.45 |
|  |  | Team sport | 7.10 ± 2.45 |
|  | Training hours | < 10 hours | 6.86 ± 2.57 |
|  |  | > 10 hours | 7.18 ± 2.36 |
|  | School program | Specialization in general studies | 7.08 ± 2.45 |
|  |  | Sports and physical education | 7.02 ± 2.45 |
|  | School level | First grade | 7.06 ± 2.38 |
|  |  | Second grade | 7.07 ± 2.52 |
|  |  | Third grade | 7.02 ± 2.47 |
| SLE | Gender | Male | 5.46 ± 2.77 |
|  |  | Female | 6.67 ± 3.26 |
|  | Type of sport | Individual | 6.33 ± 3.26 |
|  |  | Team sport | 5.89 ± 2.98 |
|  | Training hours | < 10 hours | 6.18 ± 3.16 |
|  |  | > 10 hours | 5.95 ± 3.02 |
|  | School program | Specialization in general studies | 5.94 ± 3.02 |
|  |  | Sports and physical education | 6.18 ± 3.15 |
|  | School level | First grade | 5.79 ± 2.96 |
|  |  | Second grade | 6.33 ± 3.17 |
|  |  | Third grade | 5.98 ± 3.06 |
| STR | Gender | Male | 9.91 ± 3.13 |
|  |  | Female | 11.62 ± 3.36 |
|  | Type of sport | Individual | 11.04 ± 3.58 |
|  |  | Team sport | 10.55 ± 3.22 |
|  | Training hours | < 10 hours | 10.92 ± 3.37 |
|  |  | > 10 hours | 10.60 ± 3.34 |
|  | School program | Specialization in general studies | 10.77 ± 3.37 |
|  |  | Sports and physical education | 10.67 ± 3.33 |
|  | School level | First grade | 10.55 ± 3.16 |
|  |  | Second grade | 10.84 ± 3.63 |
|  |  | Third grade | 10.82 ± 3.20 |
| FAT | Gender | Male | 7.48 ± 2.53 |
|  |  | Female | 7.89 ± 2.77 |
|  | Type of sport | Individual | 7.59 ± 2.83 |
|  |  | Team sport | 7.72 ± 2.57 |
|  | Training hours | < 10 hours | 7.96 ± 2.81 |
|  |  | > 10 hours | 7.50 ± 2.53 |
|  | School program | Specialization in general studies | 7.62 ± 2.57 |
|  |  | Sports and physical education | 7.78 ± 2.76 |
|  | School level | First grade | 7.43 ± 2.60 |
|  |  | Second grade | 7.88 ± 2.65 |
|  |  | Third grade | 7.76 ± 2.72 |
| Notes. Dep = Depression; Vig = Vigour; Sym = Physical symptoms; Sle = Sleep disturbances; Str = Stress; Fat = Fatigue; M = Mean; SD = Standard deviation. | | | |

| Table S2. Score values of the factor predictors for the different groups. | | | |
| --- | --- | --- | --- |
| Factor | Characteristics | Modalities | M ± SD |
| Dep1 | Gender | Male | 1.43 ± 0.79 |
|  |  | Female | 1.48 ±.84 |
|  | Type of sport | Individual | 1.53 ±.89 |
|  |  | Team sport | 1.42 ± 0.77 |
|  | Training hours | < 10 hours | 1.48 ±.78 |
|  |  | > 10 hours | 1.44 ± 0.84 |
|  | School program | Specialization in general studies | 1.43 ± 0.77 |
|  |  | Sports and physical education | 1.50 ± 0.86 |
|  | School level | First grade | 1.38 ± 0.75 |
|  |  | Second grade | 1.52 ± 0.83 |
|  |  | Third grade | 1.48 ± 0.86 |
| Dep2 | Gender | Male | 1.59 ± 0.84 |
|  |  | Female | 1.91 ± 1.00 |
|  | Type of sport | Individual | 1.88 ± 1.07 |
|  |  | Team sport | 1.68 ± 0.85 |
|  | Training hours | < 10 hours | 1.76 ± 0.89 |
|  |  | > 10 hours | 1.74 ± 0.96 |
|  | School program | Specialization in general studies | 1.76 ± 0.93 |
|  |  | Sports and physical education | 1.73 ± 0.93 |
|  | School level | First grade | 1.64 ± 0.86 |
|  |  | Second grade | 1.77 ± 0.95 |
|  |  | Third grade | 1.88 ± 0.99 |
| Dep3 | Gender | Male | 1.60 ± 0.81 |
|  |  | Female | 1.66 ± 0.90 |
|  | Type of sport | Individual | 1.63 ± 0.88 |
|  |  | Team sport | 1.63 ± 0.84 |
|  | Training hours | < 10 hours | 1.63 ± 0.88 |
|  |  | > 10 hours | 1.63 ± 0.84 |
|  | School program | Specialization in general studies | 1.67 ± 0.87 |
|  |  | Sports and physical education | 1.58 ± 0.83 |
|  | School level | First grade | 1.55 ± 0.81 |
|  |  | Second grade | 1.67 ± 0.88 |
|  |  | Third grade | 1.69 ± 0.88 |
| Dep4 | Gender | Male | 1.84 ± 0.98 |
|  |  | Female | 2.22 ± 1.10 |
|  | Type of sport | Individual | 2.11 ± 1.13 |
|  |  | Team sport | 1.99 ± 1.01 |
|  | Training hours | < 10 hours | 2.05 ± 1.06 |
|  |  | > 10 hours | 2.01 ± 1.05 |
|  | School program | Specialization in general studies | 2.01 ± 1.04 |
|  |  | Sports and physical education | 2.05 ± 1.08 |
|  | School level | First grade | 1.90 ± 1.04 |
|  |  | Second grade | 2.12 ± 1.10 |
|  |  | Third grade | 2.06 ± 0.99 |
| Dep5 | Gender | Male | 1.41 ± 0.83 |
|  |  | Female | 1.55 ± 0.94 |
|  | Type of sport | Individual | 1.60 ± 1.00 |
|  |  | Team sport | 1.42 ± 0.83 |
|  | Training hours | < 10 hours | 1.51 ± 0.89 |
|  |  | > 10 hours | 1.46 ± 0.89 |
|  | School program | Specialization in general studies | 1.48 ± 0.89 |
|  |  | Sports and physical education | 1.48 ± 0.89 |
|  | School level | First grade | 1.42 ± 0.85 |
|  |  | Second grade | 1.51 ± 0.88 |
|  |  | Third grade | 1.52 ± 0.96 |
| Vig1 | Gender | Male | 2.62 ± 0.96 |
|  |  | Female | 2.79 ± 1.03 |
|  | Type of sport | Individual | 2.74 ± 1.03 |
|  |  | Team sport | 2.68 ± 0.98 |
|  | Training hours | < 10 hours | 2.77 ± 2.97 |
|  |  | > 10 hours | 2.66 ± 1.01 |
|  | School program | Specialization in general studies | 2.73 ± 0.99 |
|  |  | Sports and physical education | 2.66 ± 1.00 |
|  | School level | First grade | 2.55 ± 1.05 |
|  |  | Second grade | 2.79 ± 1.03 |
|  |  | Third grade | 2.78 ± 0.84 |
| Vig2 | Gender | Male | 2.59 ± 0.95 |
|  |  | Female | 2.65 ± 0.94 |
|  | Type of sport | Individual | 2.69 ± 0.95 |
|  |  | Team sport | 2.58 ± 0.94 |
|  | Training hours | < 10 hours | 2.64 ± 0.93 |
|  |  | > 10 hours | 2.60 ± 0.96 |
|  | School program | Specialization in general studies | 2.65 ± 0.95 |
|  |  | Sports and physical education | 2.57 ± 0.95 |
|  | School level | First grade | 2.52 ± 0.98 |
|  |  | Second grade | 2.67 ± 0.98 |
|  |  | Third grade | 2.68 ± 0.84 |
| Vig3 | Gender | Male | 2.42 ± 0.87 |
|  |  | Female | 2.61 ± 0.91 |
|  | Type of sport | Individual | 2.53 ± 0.94 |
|  |  | Team sport | 2.50 ± 0.88 |
|  | Training hours | < 10 hours | 2.62 ± 0.84 |
|  |  | > 10 hours | 2.44 ± 0.92 |
|  | School program | Specialization in general studies | 2.54 ± 0.90 |
|  |  | Sports and physical education | 2.47 ± 0.89 |
|  | School level | First grade | 2.44 ± 0.92 |
|  |  | Second grade | 2.56 ± 0.90 |
|  |  | Third grade | 2.54 ± 0.85 |
| Vig4 | Gender | Male | 2.77 ± 0.95 |
|  |  | Female | 2.97 ± 0.92 |
|  | Type of sport | Individual | 2.87 ± 1.01 |
|  |  | Team sport | 2.87 ± 0.90 |
|  | Training hours | < 10 hours | 2.98 ± 0.89 |
|  |  | > 10 hours | 2.80 ± 0.96 |
|  | School program | Specialization in general studies | 2.84 ± 0.96 |
|  |  | Sports and physical education | 2.90 ± 0.90 |
|  | School level | First grade | 2.88 ± 0.99 |
|  |  | Second grade | 2.84 ± 0.91 |
|  |  | Third grade | 2.89 ± 0.90 |
| Sym1 | Gender | Male | 2.42 ± 1.05 |
|  |  | Female | 2.61 ± 1.00 |
|  | Type of sport | Individual | 2.55 ± 1.03 |
|  |  | Team sport | 2.50 ± 1.03 |
|  | Training hours | < 10 hours | 2.37 ± 1.05 |
|  |  | > 10 hours | 2.61 ± 1.01 |
|  | School program | Specialization in general studies | 2.56 ± 1.02 |
|  |  | Sports and physical education | 2.46 ± 1.04 |
|  | School level | First grade | 2.53 ± 1.03 |
|  |  | Second grade | 2.55 ± 1.05 |
|  |  | Third grade | 2.44 ± 1.01 |
| Sym2 | Gender | Male | 2.38 ± 0.98 |
|  |  | Female | 2.48 ± 0.98 |
|  | Type of sport | Individual | 2.35 ± 1.00 |
|  |  | Team sport | 2.47 ± 0.97 |
|  | Training hours | < 10 hours | 2.40 ± 0.99 |
|  |  | > 10 hours | 2.45 ± 0.97 |
|  | School program | Specialization in general studies | 2.43 ± 0.97 |
|  |  | Sports and physical education | 2.41 ± 0.99 |
|  | School level | First grade | 2.43 ± 0.97 |
|  |  | Second grade | 2.43 ± 0.99 |
|  |  | Third grade | 2.43 ± 0.98 |
| Sym3 | Gender | Male | 2.07 ± 0.99 |
|  |  | Female | 2.14 ± 1.06 |
|  | Type of sport | Individual | 2.05 ± 1.05 |
|  |  | Team sport | 2.14 ± 1.01 |
|  | Training hours | < 10 hours | 2.10 ± 1.07 |
|  |  | > 10 hours | 2.11 ± 1.00 |
|  | School program | Specialization in general studies | 2.10 ± 1.00 |
|  |  | Sports and physical education | 2.12 ± 1.06 |
|  | School level | First grade | 2.10 ± 1.00 |
|  |  | Second grade | 2.10 ± 1.02 |
|  |  | Third grade | 2.14 ± 1.07 |
| Sle1 | Gender | Male | 1.99 ± 1.13 |
|  |  | Female | 2.31 ± 1.21 |
|  | Type of sport | Individual | 2.23 ± 1.22 |
|  |  | Team sport | 2.11 ± 1.16 |
|  | Training hours | < 10 hours | 2.20 ± 1.22 |
|  |  | > 10 hours | 2.12 ± 1.16 |
|  | School program | Specialization in general studies | 2.13 ± 1.16 |
|  |  | Sports and physical education | 2.18 ± 1.21 |
|  | School level | First grade | 2.07 ± 1.16 |
|  |  | Second grade | 2.26 ± 1.21 |
|  |  | Third grade | 2.09 ± 1.16 |
| Sle2 | Gender | Male | 1.80 ± 1.00 |
|  |  | Female | 2.35 ± 1.25 |
|  | Type of sport | Individual | 2.18 ± 1.23 |
|  |  | Team sport | 2.00 ± 1.12 |
|  | Training hours | < 10 hours | 2.08 ± 1.17 |
|  |  | > 10 hours | 2.05 ± 1.16 |
|  | School program | Specialization in general studies | 2.05 ± 1.17 |
|  |  | Sports and physical education | 2.08 ± 1.16 |
|  | School level | First grade | 1.96 ± 1.13 |
|  |  | Second grade | 2.15 ± 1.18 |
|  |  | Third grade | 2.08 ± 1.17 |
| Sle3 | Gender | Male | 1.65 ± 0.98 |
|  |  | Female | 2.01 ± 1.20 |
|  | Type of sport | Individual | 1.90 ± 1.17 |
|  |  | Team sport | 1.79 ± 1.07 |
|  | Training hours | < 10 hours | 1.89 ± 1.15 |
|  |  | > 10 hours | 1.78 ± 1.08 |
|  | School program | Specialization in general studies | 1.76 ± 1.06 |
|  |  | Sports and physical education | 1.92 ± 1.17 |
|  | School level | First grade | 1.75 ± 1.05 |
|  |  | Second grade | 1.92 ± 1.18 |
|  |  | Third grade | 1.79 ± 1.08 |
| Str1 | Gender | Male | 2.75 ± 1.06 |
|  |  | Female | 3.40 ± 1.06 |
|  | Type of sport | Individual | 3.18 ± 1.18 |
|  |  | Team sport | 3.00 ± 1.07 |
|  | Training hours | < 10 hours | 3.13 ± 1.10 |
|  |  | > 10 hours | 3.02 ± 1.11 |
|  | School program | Specialization in general studies | 3.09 ± 1.11 |
|  |  | Sports and physical education | 3.02 ± 1.10 |
|  | School level | First grade | 3.02 ± 1.08 |
|  |  | Second grade | 3.10 ± 1.19 |
|  |  | Third grade | 3.07 ± 1.01 |
| Str2 | Gender | Male | 2.60 ± 1.00 |
|  |  | Female | 2.94 ± 1.02 |
|  | Type of sport | Individual | 2.79 ± 1.07 |
|  |  | Team sport | 2.75 ± 1.01 |
|  | Training hours | < 10 hours | 2.77 ± 0.99 |
|  |  | > 10 hours | 2.75 ± 1.05 |
|  | School program | Specialization in general studies | 2.73 ± 1.04 |
|  |  | Sports and physical education | 2.81 ± 1.01 |
|  | School level | First grade | 2.70 ± 0.96 |
|  |  | Second grade | 2.82 ± 1.09 |
|  |  | Third grade | 2.76 ± 1.02 |
| Str3 | Gender | Male | 1.96 ± 0.90 |
|  |  | Female | 2.28 ± 1.01 |
|  | Type of sport | Individual | 2.20 ± 1.11 |
|  |  | Team sport | 2.08 ± 0.88 |
|  | Training hours | < 10 hours | 2.17 ± 0.94 |
|  |  | > 10 hours | 2.08 ± 0.98 |
|  | School program | Specialization in general studies | 2.12 ± 0.95 |
|  |  | Sports and physical education | 2.12 ± 0.98 |
|  | School level | First grade | 2.04 ± 0.92 |
|  |  | Second grade | 2.17 ± 1.01 |
|  |  | Third grade | 2.15 ± 0.95 |
| Str4 | Gender | Male | 2.58 ± 1.05 |
|  |  | Female | 3.00 ± 1.08 |
|  | Type of sport | Individual | 2.87 ± 1.09 |
|  |  | Team sport | 2.74 ± 1.08 |
|  | Training hours | < 10 hours | 2.82 ± 1.13 |
|  |  | > 10 hours | 2.75 ± 1.05 |
|  | School program | Specialization in general studies | 2.83 ± 1.10 |
|  |  | Sports and physical education | 2.71 ± 1.05 |
|  | School level | First grade | 2.78 ± 1.06 |
|  |  | Second grade | 2.74 ± 1.15 |
|  |  | Third grade | 2.84 ± 1.02 |
| Fat1 | Gender | Male | 2.60 ± 0.99 |
|  |  | Female | 2.78 ± 0.98 |
|  | Type of sport | Individual | 2.62 ± 1.02 |
|  |  | Team sport | 2.72 ± 0.97 |
|  | Training hours | < 10 hours | 2.81 ± 1.03 |
|  |  | > 10 hours | 2.61 ± 0.95 |
|  | School program | Specialization in general studies | 2.67 ± 0.92 |
|  |  | Sports and physical education | 2.71 ± 1.07 |
|  | School level | First grade | 2.53 ± 0.94 |
|  |  | Second grade | 2.76 ± 1.01 |
|  |  | Third grade | 2.80 ± 0.99 |
| Fat2 | Gender | Male | 2.54 ± 1.06 |
|  |  | Female | 2.53 ± 1.11 |
|  | Type of sport | Individual | 2.47 ± 1.10 |
|  |  | Team sport | 2.56 ± 1.08 |
|  | Training hours | < 10 hours | 2.62 ± 1.13 |
|  |  | > 10 hours | 2.47 ± 1.05 |
|  | School program | Specialization in general studies | 2.49 ± 1.05 |
|  |  | Sports and physical education | 2.59 ± 1.14 |
|  | School level | First grade | 2.45 ± 1.07 |
|  |  | Second grade | 2.59 ± 1.13 |
|  |  | Third grade | 2.57 ± 1.04 |
| Fat3 | Gender | Male | 2.34 ± 1.00 |
|  |  | Female | 2.58 ± 1.13 |
|  | Type of sport | Individual | 2.48 ± 1.09 |
|  |  | Team sport | 2.44 ± 1.05 |
|  | Training hours | < 10 hours | 2.52 ± 1.12 |
|  |  | > 10 hours | 2.41 ± 1.03 |
|  | School program | Specialization in general studies | 2.46 ± 1.07 |
|  |  | Sports and physical education | 2.44 ± 1.06 |
|  | School level | First grade | 2.42 ± 1.03 |
|  |  | Second grade | 2.53 ± 1.09 |
|  |  | Third grade | 2.39 ± 1.09 |
| Notes. Dep1 = Miserable; Dep2 = Unhappy; Dep3 = Bitter; Dep4 = Downhearted; Dep5 = Depressed; Vig1 = Energetic; Vig2 = Lively; Vig3 = Active; Vig4 = Alert; Sym1 = Muscle soreness; Sym2 = Heavy arms or legs; Sym3 = Stiff/ sore joints; Sle1 = Difficulties falling asleep; Sle2 = Restless sleep; Sle3 = Insomnia; Str1 = Stressed; Str2 = Could not cope; Str3 = Difficulties piling up; Str4 = Nervous; Fat1 = Tired; Fat2 = Sleepy; Fat3 = Worn-out. | | | |

Results of the preliminary pilot testing

Participants

The participants in this study (n) were 162 respondents from different Counties in Norway divided between males (n = 111) and females (n = 51). The mean (M) age ± standard deviation (SD) of the participants was 17.4 ± 3.3 years old. Athletes were recruited from different sports with the majority (79.6%) from soccer, further, 5.6% from team handball, 6.2% from track and field, and 8.6% from other individual sports. Some participants combined teams- and individual sports (3.7%). Informed consent was obtained from all participants who agreed to take part in this study. The participants gave their consent by completing the electronic questionnaire. Guardians did not sign the consent.

1. Results of the preliminary pilot testing

1.1. Item analysis of MTDS-N

Of the 162 respondents included in the pilot study, there were no missing data. Table 1 presents descriptive statistics for the data. The skewness and kurtosis values ranged between 0.08–1.80 and -.06–2.81, respectively. The data were a little skewed and kurtotic, but most of the items were within the values of ±2.0, indicating approximately normally distributed data. The items *miserable* and *depressed* did not meet the criteria of ±2.0, showing kurtosis values of 2.82 and 2.47, respectively. The statistical tests KS and SW yielded statistically significant (*p* < *0*.001) results for all items, indicating not normally distributed data.

**Table 1.** Descriptive statistics for 162 participants on the items of MTDS-N.

| **Items** | **Descriptive Statistics** | | | |
| --- | --- | --- | --- | --- |
|  | **M** | **SD** | **Skewness** | **Kurtosis** |
| **Depression (dep1–dep5)** |  |  |  |  |
| Miserable (dep1) | 1.49 | 0.83 | 1.78 | 2.82 |
| Unhappy (dep2) | 1.75 | 0.92 | 1.30 | 1.39 |
| Bitter (dep3) | 1.81 | 0.98 | 1.14 | 0.66 |
| Downhearted (dep4) | 2.08 | 1.01 | 0.79 | 0.06 |
| Depressed (dep5) | 1.49 | 0.88 | 1.80 | 2.47 |
| **Vigour (vig1–vig4)** |  |  |  |  |
| Energetic (vig1) | 2.60 | 0.98 | 0.32 | −0.33 |
| Lively (vig2) | 1.50 | 0.91 | 0.46 | .14 |
| Active (vig3) | 2.55 | 0.97 | 0.44 | −0.03 |
| Alert (vig4) | 2.86 | 0.87 | 0.27 | −0.06 |
| **Physical symptoms (sym1–sym3)** |  |  |  |  |
| Muscle soreness (sym1) | 2.93 | 0.92 | 0.15 | −0.54 |
| Heaviness (sym2) | 2.60 | 1.01 | 0.28 | −0.65 |
| Joint stiffness (sym3) | 2.35 | 1.05 | 0.43 | −0.64 |
| **Sleep disturbances (sle1–sle3)** |  |  |  |  |
| Falling asleep (sle1) | 1.96 | 1.04 | 1.03 | 0.31 |
| Restless sleep (sle2) | 2.12 | 1.13 | 0.79 | −0.29 |
| Insomnia (sle3) | 1.74 | 0.98 | 1.34 | 1.28 |
| **Stress (str1–str4)** |  |  |  |  |
| Stressed (str1) | 3.01 | 1.07 | 0.08 | −0.41 |
| Cope (str2) | 2.63 | 0.97 | 0.14 | −0.24 |
| Piling (str3) | 2.00 | 0.93 | 0.88 | 0.72 |
| Nervous (str4) | 2.71 | 1.02 | 0.26 | −0.26 |
| **Fatigue (fat1–fat3)** |  |  |  |  |
| Tired (fat1) | 2.77 | 1.08 | 0.32 | −0.80 |
| Sleepy (fat2) | 2.73 | 1.11 | 0.32 | −0.80 |
| Worn out (fat3) | 2.88 | 1.14 | 0.17 | −0.80 |

M = Mean; SD = Standard deviation; Dep = Depression; Vig = Vigour; Sym = Physical symptoms; Sle = Sleep disturbances; Str = Stress; Fat = Fatigue.

To examine the extent to which athletes reported symptoms of psychophysiological stress related to training, scores from the MTDS-N were investigated. Taken collectively, as shown in Table 2, athletes' reports of training distress were moderate. Most of the subscales' (i.e., *vigour*, *physical symptoms*, *stress*, and *fatigue*) mean scores were between the range of "moderate amount" and "quite a bit." The only exception was *depression* (M = 1.73; SD = 0.92) and *sleep disturbances* (M = 1.94; SD = 1.05) scoring between “a little bit” and “moderate amount”. The total score of the six factors was 14.36 (SD = 6.01).

**Table 2.** Mean scale scores for the six factors in MTDS.

|  | **Descriptive Statistics** | |
| --- | --- | --- |
| **Factor** | **M** | **SD** |
| 1. Depression (dep) | 1.73 | 0.92 |
| 2. Vigour (vig) | 2.63 | 0.93 |
| 3. Physical symptoms (sym) | 2.63 | 1.00 |
| 4. Sleep disturbances (sle) | 1.94 | 1.05 |
| 5. Stress (str) | 2.59 | 1.00 |
| 6. Fatigue (fat) | 2.79 | 1.11 |
| Total score ^a^ | 14.31 | 6.01 |

^a^ Total score represents the sum of the six MTDS factors.

*1.2. Confirmatory factor analysis*

In the first step, a restrictive model (H0 model) were analysed, where all covariance between the six factors were fixed to zero. The results indicated a χ^2^ value of 1174.13, degrees of freedom (df) = 209, and *p* < *0*.001. None of the goodness-of-fit indices reached acceptable values: RMSEA = 0.169 (CI = 0.159–.178), CFI = 0.672, TLI = 0.638, and SRMR = 0.202.

In the second step, the six-factor solution proposed by Main and Grove (2009) were tested. This was a less restricted alternative (H1 model) compared to the H0 model. The result of the model comparison with the χ^2^ difference test revealed a *p* < *0*.001, indicating that constraining the parameters of the nested model statistically significantly worsened the fit of the model. Hence, the H1 model was preferred and retained.

The retained six-factor solution containing 22 items did not show a good fit with the data. As shown in Table 3, CFA results indicated a statistically significant χ^2^ value = 409.77, df = 194, *p* < *0*.001. The RMSEA value was 0.083, indicating a poor fit. The CFI and TLI were 0.93 and 0.92 respectively, which is below the 0.95 criterion for model acceptability. The SRMR was 0.08, which is the criterion for model acceptability.

**Table 3.** The test of model fit from the six-factor solution proposed by Main and Grove (2009) and the alternative model.

| Fit indices | The six-factor solution | The alternative model |
| --- | --- | --- |
| χ^2^ | 409.77 | 265.167 |
| df | 194 | 155 |
| *p* | <0.001 | <0.001 |
| RMSEA | 0.083 | 0.066 |
| CI | 0.07–0.09 | 0.052–0.080 |
| CFI | 0.927 | 0.961 |
| TLI | 0.913 | 0.953 |
| SRMR | 0.077 | 0.063 |

χ2 = Chi-Square Value; Df = Degree of freedom; *P* = P-value; RMSEA = Root Mean Square Error of Approximation; CI = Confidence interval; CFI = Comparative Fit Index; TLI = Tucker-Lewis Index; SRMR = Standardized Root Mean Square Residual.

1.2.1. The test for the alternative measurement model

Because the hypothesized factor model yielded a poor fit, MI was examined as a guide in search of model misspecification. Modification indices reported three relatively high measurements errors; the item *alert* (vig4) with the item *active* (vig3) = 62.65 (EPC = 0.51), the factor *physical symptoms* by the item *bitter* (dep3) = 29.55 (EPC = 0.74), and the factor *stress* by the item *bitter* (dep3) = 20.89 (EPC = −0.88). An alternative model was run, where the measurement errors were taken into consideration. Taken together, as seen in Table 3, these changes yielded a CFA result indicating a statistically significant χ^2^ = 315.25, df = 191, *p* < 0.001. The RMSEA value was 0.063, which is close to the 0.06 criteria for a good fit. The CFI and TLI were 0.96 and 0.95, respectively, both above or at the 0.95 criterion for acceptability. The SRMR was 0.067, which is below the criterion for indicating a good model. According to the χ^2^ difference test, where the MI was taken into consideration, the alternative model fitted the data statistically significantly better. The χ^2^ difference test revealed a value of *p* < 0.001, indicating that the alternative model was preferred. Standardized factor loadings and R^2^ from the hypothesized factor model and the alternative six-factor solution are provided in Table 5, while inter-factor correlations are shown in Table 6.

**Table 5.** Standardized factor loadings and R^2^ values for each item in the questionnaire for the hypothesized model and the alternative model.

| **Item** | **Hypothesized** | **R^2^** | **Alternative** | **R^2^** |
| --- | --- | --- | --- | --- |
| Miserable (dep1) | 0.888 | 0.788 | 0.884 | 0.782 |
| Unhappy (dep2) | 0.814 | 0.662 | 0.812 | 0.660 |
| Bitter (dep3) | 0.550 | 0.302 | 0.597 | 0.356 |
| Downhearted (dep4) | 0.728 | 0.530 | 0.719 | 0.517 |
| Depressed (dep5) | 0.946 | 0.896 | 0.941 | 0.886 |
| Energetic (vig1) | 0.926 | 0.858 | 0.937 | 0.877 |
| Lively (vig2) | 0.878 | 0.770 | 0.896 | 0.803 |
| Active (vig3) | 0.542 | 0.294 | 0.374 | 0.140 |
| Alert (vig4) | 0.475 | 0.226 | 0.267 | 0.071 |
| Muscle soreness (sym1) | 0.530 | 0.281 | 0.527 | 0.277 |
| Heaviness (sym2) | 0.857 | 0.734 | 0.861 | 0.742 |
| Joint stiffness (sym3) | 0.745 | 0.554 | 0.743 | 0.551 |
| Falling asleep (sle1) | 0.801 | 0.642 | 0.802 | 0.643 |
| Restless sleep (sle2) | 0.903 | 0.816 | 0.903 | 0.816 |
| Insomnia (sle3) | 0.908 | 0.824 | 0.908 | 0.824 |
| Stressed (str1) | 0.768 | 0.590 | 0.768 | 0.590 |
| Cope (str2) | 0.731 | 0.535 | 0.731 | 0.535 |
| Piling (str3) | 0.784 | 0.615 | 0.788 | 0.621 |
| Nervous (str4) | 0.756 | 0.572 | 0.753 | 0.567 |
| Tired (fat1) | 0.768 | 0.590 | 0.769 | 0.592 |
| Sleepy (fat2) | 0.745 | 0.555 | 0.743 | 0.552 |
| Worn out (fat3) | 0.852 | 0.726 | 0.853 | 0.727 |

R^2^ = Coefficient of Determination.

**Table 6.** Standardized inter-factor correlations from the alternative model above the diagonal (in **Bold**) and inter-correlations from the initial study of MTDS are presented below the diagonal.

| **Factor** | **Depression** | **Vigour** | **Physical Symptoms** | **Sleep disturbances** | **Stress** | **Fatigue** |
| --- | --- | --- | --- | --- | --- | --- |
| DEP | 1 | **−0.210 *** | **0.101** | **0.441**** | **0.777 **** | **0.632 **** |
| VIG | −0.194 | 1 | **−0.159** | **−0.227*** | **−0.143** | **−0.238 *** |
| SYM | −0.228 | 0.041 | 1 | **0.269**** | **0.019** | **0.470 **** |
| SLE | −0.394 | 0.110 | 0.247 | 1 | **0.271 **** | **0.484 **** |
| STR | 0.437 | −0.259 | −0.181 | −0.273 | 1 | **0.495 **** |
| FAT | −0.208 | 0.182 | 0.321 | 0.207 | -.311 | 1 |

* = *p* < *0*.05; ** = *p* < *0*.001.


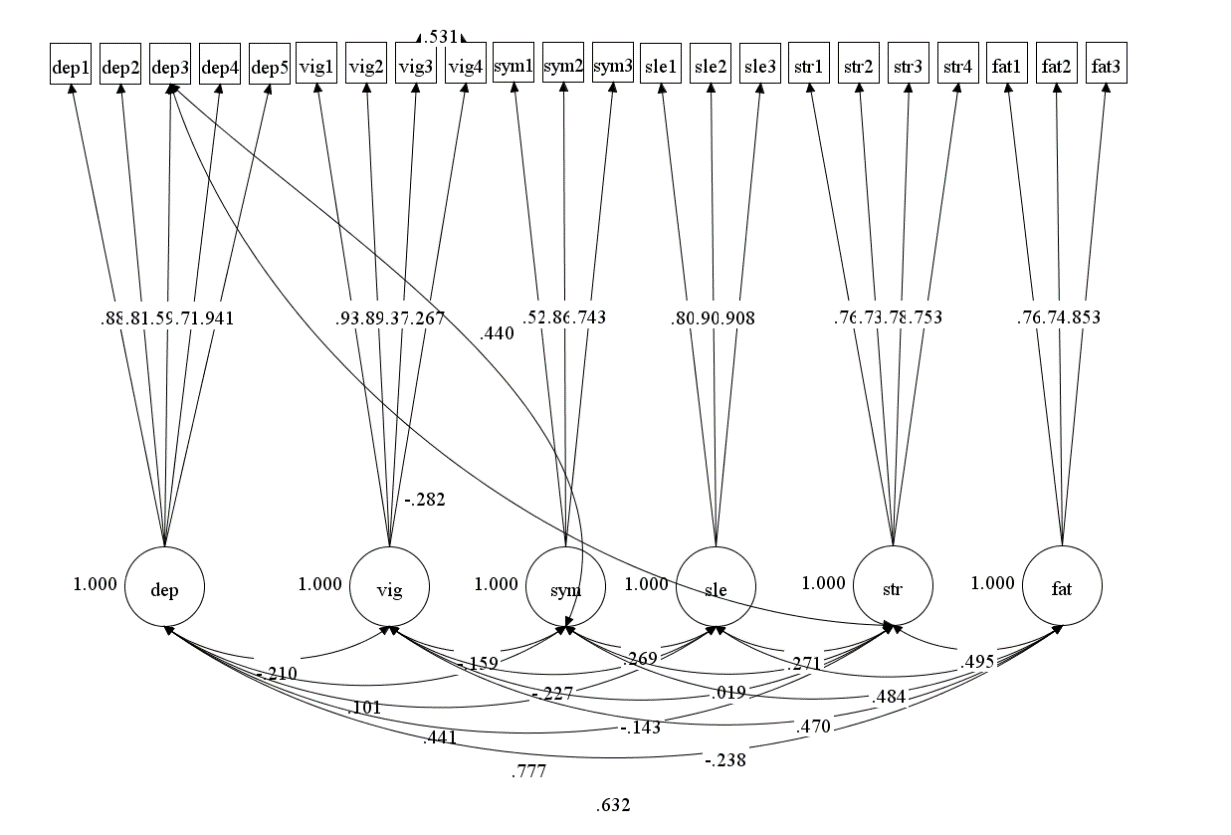


**Figure 1.** Standardized factor loadings and covariance estimates from the alternative model.

As presented in figure 1, all standardized factor loadings were statistically significant (*p* < *0*.001) and in the expected direction, ranging from 0.267–0.941. The high loadings in the measurement model indicate a strong association between each of the latent factors and their respective items. Average factor loadings for *depression, vigour, physical symptoms, sleep disturbances, stress,* and *fatigue* were 0.791, 0.619, 0.710, 0.871, 0.760, and 0.788, respectively. Average factor loadings were all above the average R^2^ value (.640, 0.473, 0.523, 0.761, 0.578, and 0.624, respectively).

3.3. Reliability analysis

Internal consistency of all factors were: α = 0.83 for factor 1 *depression*, α = 0.72 for factor 2 *vigour*, α = 0.72 for factor 3 *physical symptoms*, α = 0.87 for factor 4 *sleep disturbances*, α 0.80 for factor 5 *stress*, and α = 0.80 for factor 6 *fatigue*.
