# Supplementary material for: The Factorial Validity of the Norwegian Version of the Multicomponent Training Distress Scale (MTDS-N)
Source: Int J Environ Res Public Health. 2020 Oct 19;17(20):7603. doi: 10.3390/ijerph17207603 (PMC7590227; doi:10.3390/ijerph17207603)
Supplement: Supplementary file 1 [file ijerph-17-07603-s001.zip › ijerph-944040-Supplementary/Supplementary Materials.pdf]

Supplementary

Table S1. Score values of the factors for the different groups.

Factor	Characteristics	Modalities	M ± SD
DEP	Gender	Male	7.90 ± 3.38
		Female	8.83 ± 3.88
	Type of sport	Individual	8.83 ± 4.23
		Team sport	8.14 ± 3.40
	Training hours	< 10 hours	8.45 ± 3.58
		> 10 hours	8.33 ± 3.79
	School program	Specialization in general studies	8.35 ± 3.59
		Sports and physical education	8.41 ± 3.88
	School level	First grade	7.96 ± 3.53
		Second grade	8.59 ± 3.83
		Third grade	8.66 ± 3.74
VIG	Gender	Male	10.41 ± 2.81
		Female	11.01 ± 2.96
	Type of sport	Individual	10.83 ± 3.09
		Team sport	10.63 ± 2.80
	Training hours	< 10 hours	11.00 ± 2.72
		> 10 hours	10.50 ± 2.98
	School program	Specialization in general studies	10.77 ± 2.93
		Sports and physical education	10.60 ± 2.83
	School level	First grade	10.39 ± 2.96
		Second grade	10.86 ± 3.03
		Third grade	10.89 ± 2.53
SYM	Gender	Male	6.88 ± 2.47
		Female	7.23 ± 2.42
	Type of sport	Individual	6.97 ± 2.45
		Team sport	7.10 ± 2.45
	Training hours	< 10 hours	6.86 ± 2.57
		> 10 hours	7.18 ± 2.36
	School program	Specialization in general studies	7.08 ± 2.45
		Sports and physical education	7.02 ± 2.45
	School level	First grade	7.06 ± 2.38
		Second grade	7.07 ± 2.52
		Third grade	7.02 ± 2.47
SLE	Gender	Male	5.46 ± 2.77
		Female	6.67 ± 3.26
	Type of sport	Individual	6.33 ± 3.26
		Team sport	5.89 ± 2.98
	Training hours	< 10 hours	6.18 ± 3.16
		> 10 hours	5.95 ± 3.02

	School program	Specialization in general studies	5.94 ± 3.02
		Sports and physical education	6.18 ± 3.15
	School level	First grade	5.79 ± 2.96
		Second grade	6.33 ± 3.17
		Third grade	5.98 ± 3.06
STR	Gender	Male	9.91 ± 3.13
		Female	11.62 ± 3.36
	Type of sport	Individual	11.04 ± 3.58
		Team sport	10.55 ± 3.22
	Training hours	< 10 hours	10.92 ± 3.37
		> 10 hours	10.60 ± 3.34
	School program	Specialization in general studies	10.77 ± 3.37
		Sports and physical education	10.67 ± 3.33
	School level	First grade	10.55 ± 3.16
		Second grade	10.84 ± 3.63
		Third grade	10.82 ± 3.20
FAT	Gender	Male	7.48 ± 2.53
		Female	7.89 ± 2.77
	Type of sport	Individual	7.59 ± 2.83
		Team sport	7.72 ± 2.57
	Training hours	< 10 hours	7.96 ± 2.81
		> 10 hours	7.50 ± 2.53
	School program	Specialization in general studies	7.62 ± 2.57
		Sports and physical education	7.78 ± 2.76
	School level	First grade	7.43 ± 2.60
		Second grade	7.88 ± 2.65
		Third grade	7.76 ± 2.72

Notes. Dep = Depression; Vig = Vigour; Sym = Physical symptoms; Sle = Sleep disturbances; Str = Stress; Fat = Fatigue; M = Mean; SD = Standard deviation.

Table S2. Score values of the factor predictors for the different groups.

Factor	Characteristics	Modalities	M ± SD
Dep1	Gender	Male	1.43 ± .79
		Female	1.48 ±.84
	Type of sport	Individual	1.53 ±.89
		Team sport	1.42 ± .77
	Training hours	< 10 hours	1.48 ±.78
		> 10 hours	1.44 ± .84
	School program	Specialization in general studies	1.43 ± .77
		Sports and physical education	1.50 ± .86
	School level	First grade	1.38 ± .75
		Second grade	1.52 ± .83
		Third grade	1.48 ± .86
Dep2	Gender	Male	1.59 ± .84
		Female	1.91 ± 1.00
	Type of sport	Individual	1.88 ± 1.07
		Team sport	1.68 ± .85
	Training hours	< 10 hours	1.76 ± .89
		> 10 hours	1.74 ± .96
	School program	Specialization in general studies	1.76 ± .93
		Sports and physical education	1.73 ± .93
	School level	First grade	1.64 ± .86
		Second grade	1.77 ± .95
		Third grade	1.88 ± .99
Dep3	Gender	Male	1.60 ± .81
		Female	1.66 ± .90
	Type of sport	Individual	1.63 ± .88
		Team sport	1.63 ± .84
	Training hours	< 10 hours	1.63 ± .88
		> 10 hours	1.63 ± .84
	School program	Specialization in general studies	1.67 ± .87
		Sports and physical education	1.58 ± .83
	School level	First grade	1.55 ± .81
		Second grade	1.67 ± .88
		Third grade	1.69 ± .88
Dep4	Gender	Male	1.84 ± .98
		Female	2.22 ± 1.10
	Type of sport	Individual	2.11 ± 1.13
		Team sport	1.99 ± 1.01
	Training hours	< 10 hours	2.05 ± 1.06
		> 10 hours	2.01 ± 1.05
	School program	Specialization in general studies	2.01 ± 1.04
		Sports and physical education	2.05 ± 1.08

	School level	First grade	1.90 ± 1.04
		Second grade	2.12 ± 1.10
		Third grade	2.06 ± .99
Dep5	Gender	Male	1.41 ± .83
		Female	1.55 ± .94
	Type of sport	Individual	1.60 ± 1.00
		Team sport	1.42 ± .83
	Training hours	< 10 hours	1.51 ± .89
		> 10 hours	1.46 ± .89
	School program	Specialization in general studies	1.48 ± .89
		Sports and physical education	1.48 ± .89
	School level	First grade	1.42 ± .85
		Second grade	1.51 ± .88
		Third grade	1.52 ± .96
Vig1	Gender	Male	2.62 ± .96
		Female	2.79 ± 1.03
	Type of sport	Individual	2.74 ± 1.03
		Team sport	2.68 ± .98
	Training hours	< 10 hours	2.77 ± 2.97
		> 10 hours	2.66 ± 1.01
	School program	Specialization in general studies	2.73 ± .99
		Sports and physical education	2.66 ± 1.00
	School level	First grade	2.55 ± 1.05
		Second grade	2.79 ± 1.03
		Third grade	2.78 ± .84
Vig2	Gender	Male	2.59 ± .95
		Female	2.65 ± .94
	Type of sport	Individual	2.69 ± .95
		Team sport	2.58 ± .94
	Training hours	< 10 hours	2.64 ± .93
		> 10 hours	2.60 ± .96
	School program	Specialization in general studies	2.65 ± .95
		Sports and physical education	2.57 ± .95
	School level	First grade	2.52 ± .98
		Second grade	2.67 ± .98
		Third grade	2.68 ± .84
Vig3	Gender	Male	2.42 ± .87
		Female	2.61 ± .91
	Type of sport	Individual	2.53 ± .94
		Team sport	2.50 ± .88
	Training hours	< 10 hours	2.62 ± .84
		> 10 hours	2.44 ± .92
	School program	Specialization in general studies	2.54 ± .90

		Sports and physical education	2.47 ± .89
	School level	First grade	2.44 ± .92
		Second grade	2.56 ± .90
		Third grade	2.54 ± .85
Vig4	Gender	Male	2.77 ± .95
		Female	2.97 ± .92
	Type of sport	Individual	2.87 ± 1.01
		Team sport	2.87 ± .90
	Training hours	< 10 hours	2.98 ± .89
		> 10 hours	2.80 ± .96
	School program	Specialization in general studies	2.84 ± .96
		Sports and physical education	2.90 ± .90
	School level	First grade	2.88 ± .99
		Second grade	2.84 ± .91
		Third grade	2.89 ± .90
Sym1	Gender	Male	2.42 ± 1.05
		Female	2.61 ± 1.00
	Type of sport	Individual	2.55 ± 1.03
		Team sport	2.50 ± 1.03
	Training hours	< 10 hours	2.37 ± 1.05
		> 10 hours	2.61 ± 1.01
	School program	Specialization in general studies	2.56 ± 1.02
		Sports and physical education	2.46 ± 1.04
	School level	First grade	2.53 ± 1.03
		Second grade	2.55 ± 1.05
		Third grade	2.44 ± 1.01
Sym2	Gender	Male	2.38 ± .98
		Female	2.48 ± .98
	Type of sport	Individual	2.35 ± 1.00
		Team sport	2.47 ± .97
	Training hours	< 10 hours	2.40 ± .99
		> 10 hours	2.45 ± .97
	School program	Specialization in general studies	2.43 ± .97
		Sports and physical education	2.41 ± .99
	School level	First grade	2.43 ± .97
		Second grade	2.43 ± .99
		Third grade	2.43 ± .98
Sym3	Gender	Male	2.07 ± .99
		Female	2.14 ± 1.06
	Type of sport	Individual	2.05 ± 1.05
		Team sport	2.14 ± 1.01
	Training hours	< 10 hours	2.10 ± 1.07
		> 10 hours	2.11 ± 1.00

	School program	Specialization in general studies	2.10 ± 1.00
		Sports and physical education	2.12 ± 1.06
	School level	First grade	2.10 ± 1.00
		Second grade	2.10 ± 1.02
		Third grade	2.14 ± 1.07
Sle1	Gender	Male	1.99 ± 1.13
		Female	2.31 ± 1.21
	Type of sport	Individual	2.23 ± 1.22
		Team sport	2.11 ± 1.16
	Training hours	< 10 hours	2.20 ± 1.22
		> 10 hours	2.12 ± 1.16
	School program	Specialization in general studies	2.13 ± 1.16
		Sports and physical education	2.18 ± 1.21
	School level	First grade	2.07 ± 1.16
		Second grade	2.26 ± 1.21
		Third grade	2.09 ± 1.16
Sle2	Gender	Male	1.80 ± 1.00
		Female	2.35 ± 1.25
	Type of sport	Individual	2.18 ± 1.23
		Team sport	2.00 ± 1.12
	Training hours	< 10 hours	2.08 ± 1.17
		> 10 hours	2.05 ± 1.16
	School program	Specialization in general studies	2.05 ± 1.17
		Sports and physical education	2.08 ± 1.16
	School level	First grade	1.96 ± 1.13
		Second grade	2.15 ± 1.18
		Third grade	2.08 ± 1.17
Sle3	Gender	Male	1.65 ± .98
		Female	2.01 ± 1.20
	Type of sport	Individual	1.90 ± 1.17
		Team sport	1.79 ± 1.07
	Training hours	< 10 hours	1.89 ± 1.15
		> 10 hours	1.78 ± 1.08
	School program	Specialization in general studies	1.76 ± 1.06
		Sports and physical education	1.92 ± 1.17
	School level	First grade	1.75 ± 1.05
		Second grade	1.92 ± 1.18
		Third grade	1.79 ± 1.08
	Gender	Male	2.75 ± .1.06
		Female	3.40 ± 1.06
	Type of sport	Individual	3.18 ± 1.18
		Team sport	3.00 ± 1.07

Str1	Training hours	< 10 hours	3.13 ± 1.10
		> 10 hours	3.02 ± 1.11
	School program	Specialization in general studies	3.09 ± 1.11
		Sports and physical education	3.02 ± 1.10
	School level	First grade	3.02 ± 1.08
		Second grade	3.10 ± 1.19
		Third grade	3.07 ± 1.01
Str2	Gender	Male	2.60 ± 1.00
		Female	2.94 ± .1.02
	Type of sport	Individual	2.79 ± 1.07
		Team sport	2.75 ± 1.01
	Training hours	< 10 hours	2.77 ± .99
		> 10 hours	2.75 ± 1.05
	School program	Specialization in general studies	2.73 ± 1.04
		Sports and physical education	2.81 ± 1.01
	School level	First grade	2.70 ± .96
		Second grade	2.82 ± 1.09
		Third grade	2.76 ± 1.02
Str3	Gender	Male	1.96 ± .90
		Female	2.28 ± 1.01
	Type of sport	Individual	2.20 ± 1.11
		Team sport	2.08 ± .88
	Training hours	< 10 hours	2.17 ± .94
		> 10 hours	2.08 ± .98
	School program	Specialization in general studies	2.12 ± .95
		Sports and physical education	2.12 ± .98
	School level	First grade	2.04 ± .92
		Second grade	2.17 ± 1.01
		Third grade	2.15 ± .95
Str4	Gender	Male	2.58 ± 1.05
		Female	3.00 ± 1.08
	Type of sport	Individual	2.87 ± 1.09
		Team sport	2.74 ± 1.08
	Training hours	< 10 hours	2.82 ± 1.13
		> 10 hours	2.75 ± 1.05
	School program	Specialization in general studies	2.83 ± 1.10
		Sports and physical education	2.71 ± 1.05
	School level	First grade	2.78 ± 1.06
		Second grade	2.74 ± 1.15
		Third grade	2.84 ± 1.02
	Gender	Male	2.60 ± .99
		Female	2.78 ± .98

Fat1	Type of sport	Individual	2.62 ± 1.02
		Team sport	2.72 ± .97
	Training hours	< 10 hours	2.81 ± 1.03
		> 10 hours	2.61 ± .95
	School program	Specialization in general studies	2.67 ± .92
		Sports and physical education	2.71 ± 1.07
	School level	First grade	2.53 ± .94
		Second grade	2.76 ± 1.01
		Third grade	2.80 ± .99
Fat2	Gender	Male	2.54 ± 1.06
		Female	2.53 ± 1.11
	Type of sport	Individual	2.47 ± 1.10
		Team sport	2.56 ± 1.08
	Training hours	< 10 hours	2.62 ± 1.13
		> 10 hours	2.47 ± 1.05
	School program	Specialization in general studies	2.49 ± 1.05
		Sports and physical education	2.59 ± 1.14
	School level	First grade	2.45 ± 1.07
		Second grade	2.59 ± 1.13
		Third grade	2.57 ± 1.04
Fat3	Gender	Male	2.34 ± 1.00
		Female	2.58 ± 1.13
	Type of sport	Individual	2.48 ± 1.09
		Team sport	2.44 ± 1.05
	Training hours	< 10 hours	2.52 ± 1.12
		> 10 hours	2.41 ± 1.03
	School program	Specialization in general studies	2.46 ± 1.07
		Sports and physical education	2.44 ± 1.06
	School level	First grade	2.42 ± 1.03
		Second grade	2.53 ± 1.09
		Third grade	2.39 ± 1.09

Notes. Dep1 = Miserable; Dep2 = Unhappy; Dep3 = Bitter; Dep4 = Downhearted; Dep5 = Depressed; Vig1 = Energetic; Vig2 = Lively; Vig3 = Active; Vig4 = Alert; Sym1 = Muscle soreness; Sym2 = Heavy arms or legs; Sym3 = Stiff/ sore joints; Sle1 = Difficulties falling asleep; Sle2 = Restless sleep; Sle3 = Insomnia; Str1 = Stressed; Str2 = Could not cope; Str3 = Difficulties piling up; Str4 = Nervous; Fat1 = Tired; Fat2 = Sleepy; Fat3 = Worn-out.

Results of the preliminary pilot testing

Participants

The participants in this study (n) were 162 respondents from different Counties in Norway divided between males (n = 111) and females (n = 51). The mean (M) age ± standard deviation (SD) of the participants was 17.4 ± 3.3 years old. Athletes were recruited from different sports with the majority (79.6%) from soccer, further, 5.6% from team handball, 6.2% from track and field, and 8.6% from other individual sports. Some participants combined teams- and individual sports (3.7%). Informed consent was obtained from all participants who agreed to take part in this study. The participants gave their consent by completing the electronic questionnaire. Guardians did not sign the consent.

1. Results of the preliminary pilot testing

1.1. Item analysis of MTDS-N

Of the 162 respondents included in the pilot study, there were no missing data. Table 1 presents descriptive statistics for the data. The skewness and kurtosis values ranged between .08–1.80 and -.06–2.81, respectively. The data were a little skewed and kurtotic, but most of the items were within the values of ±2.0, indicating approximately normally distributed data. The items *miserable* and *depressed* did not meet the criteria of ±2.0, showing kurtosis values of 2.82 and 2.47, respectively. The statistical tests KS and SW yielded statistically significant (p<.001) results for all items, indicating not normally distributed data.

Table 1. Descriptive statistics for 162 participants on the items of MTDS-N.

Items	Descriptive Statistics			
	M	SD	Skewness	Kurtosis
**Depression (dep1–dep5)**				
Miserable (dep1)	1.49	.83	1.78	2.82
Unhappy (dep2)	1.75	.92	1.30	1.39
Bitter (dep3)	1.81	.98	1.14	.66
Downhearted (dep4)	2.08	1.01	.79	.06
Depressed (dep5)	1.49	.88	1.80	2.47
**Vigour (vig1–vig4)**				
Energetic (vig1)	2.60	.98	.32	-.33
Lively (vig2)	1.50	.91	.46	.14
Active (vig3)	2.55	.97	.44	-.03
Alert (vig4)	2.86	.87	.27	-.06
**Physical symptoms (sym1–sym3)**				
Muscle soreness (sym1)	2.93	.92	.15	-.54
Heaviness (sym2)	2.60	1.01	.28	-.65
Joint stiffness (sym3)	2.35	1.05	.43	-.64
**Sleep disturbances (sle1–sle3)**				
Falling asleep (sle1)	1.96	1.04	1.03	.31
Restless sleep (sle2)	2.12	1.13	.79	-.29
Insomnia (sle3)	1.74	.98	1.34	1.28
**Stress (str1–str4)**				
Stressed (str1)	3.01	1.07	.08	-.41
Cope (str2)	2.63	.97	.14	-.24
Piling (str3)	2.00	.93	.88	.72
Nervous (str4)	2.71	1.02	.26	-.26
**Fatigue (fat1–fat3)**				
Tired (fat1)	2.77	1.08	.32	-.80
Sleepy (fat2)	2.73	1.11	.32	-.80
Worn out (fat3)	2.88	1.14	.17	-.80

M = Mean; SD = Standard deviation; Dep = Depression; Vig = Vigour; Sym = Physical symptoms; Sle = Sleep disturbances; Str = Stress; Fat = Fatigue.

To examine the extent to which athletes reported symptoms of psychophysiological stress related to training, scores from the MTDS-N were investigated. Taken collectively, as shown in Table 2, athletes' reports of training distress were moderate. Most of the subscales' (i.e., *vigour*, *physical symptoms*, *stress*,

and *fatigue*) mean scores were between the range of "moderate amount" and "quite a bit." The only exception was *depression* (M = 1.73; SD = .92) and *sleep disturbances* (M = 1.94; SD = 1.05) scoring between "a little bit" and "moderate amount". The total score of the six factors was 14.36 (SD = 6.01).

Table 2. Mean scale scores for the six factors in MTDS.

	Descriptive Statistics	
**Factor**	**M**	**SD**
1. Depression (dep)	1.73	.92
2. Vigour (vig)	2.63	.93
3. Physical symptoms (sym)	2.63	1.00
4. Sleep disturbances (sle)	1.94	1.05
5. Stress (str)	2.59	1.00
6. Fatigue (fat)	2.79	1.11
Total score[a]	14.31	6.01

[a] Total score represents the sum of the six MTDS factors.

1.2. Confirmatory factor analysis

In the first step, a restrictive model (H0 model) were analysed, where all covariance between the six factors were fixed to zero. The results indicated a χ^2 value of 1174.13, degrees of freedom (df) = 209, and $p<.001$. None of the goodness-of-fit indices reached acceptable values: RMSEA = .169 (CI = .159–.178), CFI = .672, TLI = .638, and SRMR = .202.

In the second step, the six-factor solution proposed by Main and Grove (2009) were tested. This was a less restricted alternative (H1 model) compared to the H0 model. The result of the model comparison with the χ^2 difference test revealed a $p<.001$, indicating that constraining the parameters of the nested model statistically significantly worsened the fit of the model. Hence, the H1 model was preferred and retained.

The retained six-factor solution containing 22 items did not show a good fit with the data. As shown in Table 3, CFA results indicated a statistically significant χ^2 value = 409.77, df = 194, $p<.001$. The RMSEA value was .083, indicating a poor fit. The CFI and TLI were .93 and .92 respectively, which is below the .95 criterion for model acceptability. The SRMR was .08, which is the criterion for model acceptability.

Table 3. The test of model fit from the six-factor solution proposed by Main and Grove (2009) and the alternative model.

Fit indices	The six-factor solution	The alternative model
χ^2	409.77	265.167
df	194	155
p	<.001	<.001
RMSEA	.083	.066
CI	.07–.09	.052–.080
CFI	.927	.961
TLI	.913	.953
SRMR	.077	.063

χ2 = Chi-Square Value; Df = Degree of freedom; *P* = P-value; RMSEA = Root Mean Square Error of Approximation; CI = Confidence interval; CFI = Comparative Fit Index; TLI = Tucker-Lewis Index; SRMR = Standardized Root Mean Square Residual.

1.2.1. The test for the alternative measurement model

Because the hypothesized factor model yielded a poor fit, MI was examined as a guide in search of model misspecification. Modification indices reported three relatively high measurements errors; the item *alert* (vig4) with the item *active* (vig3) = 62.65 (EPC=.51), the factor *physical symptoms* by the item *bitter* (dep3) = 29.55 (EPC=.74), and the factor *stress* by the item *bitter* (dep3) = 20.89 (EPC=-.88). An alternative model was run, where the measurement errors were taken into consideration. Taken together, as seen in Table 3, these changes yielded a CFA result indicating a statistically significant χ^2 = 315.25, df = 191, *p*<.001. The RMSEA value was .063, which is close to the .06 criteria for a good fit. The CFI and TLI were .96 and .95, respectively, both above or at the .95 criterion for acceptability. The SRMR was .067, which is below the criterion for indicating a good model. According to the χ^2 difference test, where the MI was taken into consideration, the alternative model fitted the data statistically significantly better. The χ^2 difference test revealed a value of *p*<.001, indicating that the alternative model was preferred. Standardized factor loadings and R^2 from the hypothesized factor model and the alternative six-factor solution are provided in Table 5, while inter-factor correlations are shown in Table 6.

Table 5. Standardized factor loadings and R^2 values for each item in the questionnaire for the hypothesized model and the alternative model.

Item	Hypothesized	R^2	Alternative	R^2
Miserable (dep1)	.888	.788	.884	.782
Unhappy (dep2)	.814	.662	.812	.660
Bitter (dep3)	.550	.302	.597	.356
Downhearted (dep4)	.728	.530	.719	.517
Depressed (dep5)	.946	.896	.941	.886
Energetic (vig1)	.926	.858	.937	.877
Lively (vig2)	.878	.770	.896	.803
Active (vig3)	.542	.294	.374	.140
Alert (vig4)	.475	.226	.267	.071
Muscle soreness (sym1)	.530	.281	.527	.277
Heaviness (sym2)	.857	.734	.861	.742
Joint stiffness (sym3)	.745	.554	.743	.551
Falling asleep (sle1)	.801	.642	.802	.643
Restless sleep (sle2)	.903	.816	.903	.816
Insomnia (sle3)	.908	.824	.908	.824
Stressed (str1)	.768	.590	.768	.590
Cope (str2)	.731	.535	.731	.535
Piling (str3)	.784	.615	.788	.621
Nervous (str4)	.756	.572	.753	.567
Tired (fat1)	.768	.590	.769	.592
Sleepy (fat2)	.745	.555	.743	.552
Worn out (fat3)	.852	.726	.853	.727

R^2 = Coefficient of Determination.

Table 6. Standardized inter-factor correlations from the alternative model above the diagonal (in **Bold**) and inter-correlations from the initial study of MTDS are presented below the diagonal.

Factor	Depression	Vigour	Physical Symptoms	Sleep disturbances	Stress	Fatigue
DEP	1	**-.210***	**.101**	**.441****	**.777****	**.632****
VIG	-.194	1	**-.159**	**-.227***	**-.143**	**-.238***
SYM	-.228	.041	1	**.269****	**.019**	**.470****
SLE	-.394	.110	.247	1	**.271****	**.484****
STR	.437	-.259	-.181	-.273	1	**.495****
FAT	-.208	.182	.321	.207	-.311	1

* = p<.05; ** = p<.001.

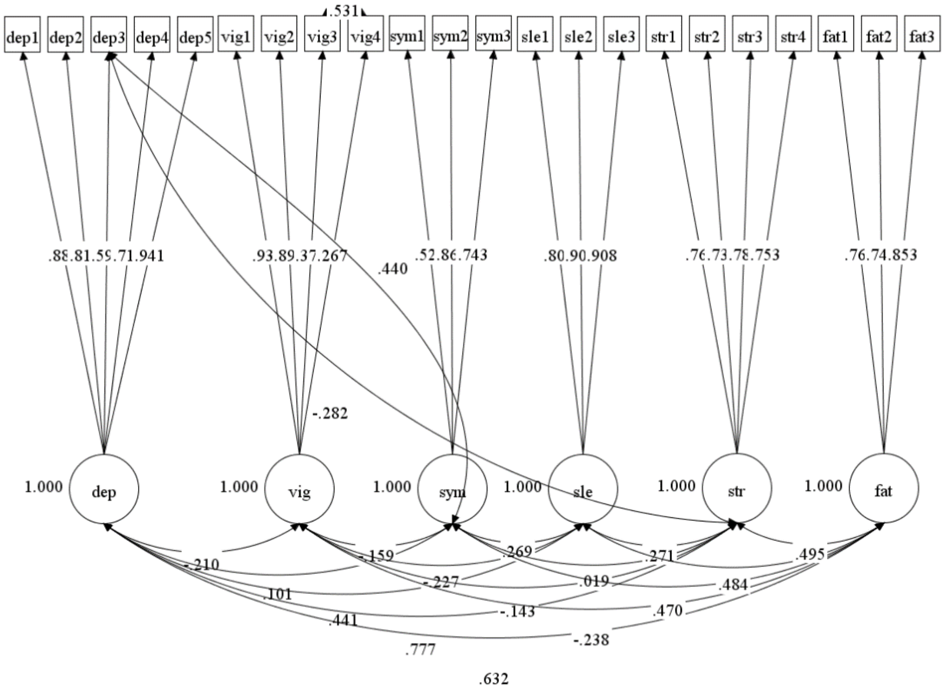

Figure 1. Standardized factor loadings and covariance estimates from the alternative model.

As presented in figure 1, all standardized factor loadings were statistically significant (*p*<.001) and in the expected direction, ranging from .267–.941. The high loadings in the measurement model indicate a strong association between each of the latent factors and their respective items. Average factor loadings for *depression, vigour, physical symptoms, sleep disturbances, stress,* and *fatigue* were .791, .619, .710, .871, .760, and .788, respectively. Average factor loadings were all above the average R^2 value (.640, .473, .523, .761, .578, and .624, respectively).

3.3. Reliability analysis

Internal consistency of all factors were: α = .83 for factor 1 *depression*, α = .72 for factor 2 *vigour*, α = .72 for factor 3 *physical symptoms*, α = .87 for factor 4 *sleep disturbances*, α .80 for factor 5 *stress*, and α = .80 for factor 6 *fatigue*.

Results of the preliminary pilot testing.
New model with "BY" statement


```
Mplus VERSION 8.4
MUTHEN & MUTHEN
09/29/2020   1:00 PM

INPUT INSTRUCTIONS

   TITLE:  CFA with M.I;
        DATA:   FILE IS  MTDS to Mplus.dat;
        VARIABLE: NAMES ARE
                  DEP_1 DEP_2 DEP_3 DEP_4 DEP_5
                  VIG_1 VIG_2 VIG_3 VIG_4
                  SYM_1 SYM_2 SYM_3
                  SLE_1 SLE_2 SLE_3
                  STR_1 STR_2 STR_3 STR_4
                  FAT_1 FAT_2 FAT_3;

                  CATEGORICAL ARE
                  DEP_1 DEP_2 DEP_3 DEP_4 DEP_5
                  VIG_1 VIG_2 VIG_3 VIG_4
                  SYM_1 SYM_2 SYM_3
                  SLE_1 SLE_2 SLE_3
                  STR_1 STR_2 STR_3 STR_4
                  FAT_1 FAT_2 FAT_3;

    ANALYSIS: PARAMETERIZATION=THETA;

    MODEL: DEP by DEP_1* DEP_2 DEP_3 DEP_4 DEP_5;
           VIG by VIG_1* VIG_2 VIG_3 VIG_4;
           SYM by SYM_1* SYM_2 SYM_3;
           SLE by SLE_1* SLE_2 SLE_3;
           STR by STR_1* STR_2 STR_3 STR_4;
           FAT by FAT_1* FAT_2 FAT_3;

           DEP@1;
           VIG@1;
           SYM@1;
           SLE@1;
           STR@1;
           FAT@1;

           VIG_4 with VIG_3; !M.I. 62.65
           SYM with DEP_3;   !M.I. 29.55
           SYM by DEP_3;
           STR with DEP_3;   !M.I. 20.89
           STR by DEP_3;
           ! Comment from reviewer: include crossloading with BY statement




  OUTPUT:
  SAMPSTAT STANDARDIZED RESIDUAL MODINDICES (ALL) ;




INPUT READING TERMINATED NORMALLY



CFA with M.I;

SUMMARY OF ANALYSIS

Number of groups                                                 1
Number of observations                                         162
```

```
Number of dependent variables                                 22
Number of independent variables                                0
Number of continuous latent variables                          6

Observed dependent variables

  Binary and ordered categorical (ordinal)
   DEP_1       DEP_2       DEP_3       DEP_4       DEP_5       VIG_1
   VIG_2       VIG_3       VIG_4       SYM_1       SYM_2       SYM_3
   SLE_1       SLE_2       SLE_3       STR_1       STR_2       STR_3
   STR_4       FAT_1       FAT_2       FAT_3

Continuous latent variables
   DEP         VIG         SYM         SLE         STR         FAT


Estimator                                                  WLSMV
Maximum number of iterations                                1000
Convergence criterion                                  0.500D-04
Maximum number of steepest descent iterations                 20
Parameterization                                           THETA
Link                                                      PROBIT

Input data file(s)
  MTDS to Mplus.dat

Input data format  FREE


UNIVARIATE PROPORTIONS AND COUNTS FOR CATEGORICAL VARIABLES

    DEP_1
      Category 1    0.673          109.000
      Category 2    0.204           33.000
      Category 3    0.086           14.000
      Category 4    0.031            5.000
      Category 5    0.006            1.000
    DEP_2
      Category 1    0.494           80.000
      Category 2    0.340           55.000
      Category 3    0.105           17.000
      Category 4    0.049            8.000
      Category 5    0.012            2.000
    DEP_3
      Category 1    0.481           78.000
      Category 2    0.315           51.000
      Category 3    0.123           20.000
      Category 4    0.068           11.000
      Category 5    0.012            2.000
    DEP_4
      Category 1    0.327           53.000
      Category 2    0.389           63.000
      Category 3    0.179           29.000
      Category 4    0.086           14.000
      Category 5    0.019            3.000
    DEP_5
      Category 1    0.704          114.000
      Category 2    0.154           25.000
      Category 3    0.093           15.000
      Category 4    0.043            7.000
      Category 5    0.006            1.000
    VIG_1
      Category 1    0.031            5.000
      Category 2    0.148           24.000
      Category 3    0.327           53.000
      Category 4    0.377           61.000
      Category 5    0.117           19.000
```

FAT_2	0.161	0.190	0.170	0.340	0.399
FAT_3	0.393	0.286	0.335	0.455	0.358

CORRELATION MATRIX (WITH VARIANCES ON THE DIAGONAL)

	STR_1	STR_2	STR_3	STR_4	FAT_1
STR_2	0.461				
STR_3	0.517	0.690			
STR_4	0.653	0.506	0.532		
FAT_1	0.241	0.239	0.190	0.229	
FAT_2	0.225	0.327	0.379	0.281	0.638
FAT_3	0.418	0.343	0.352	0.298	0.630

CORRELATION MATRIX (WITH VARIANCES ON THE DIAGONAL)

	FAT_2	FAT_3
FAT_3	0.586	

THE MODEL ESTIMATION TERMINATED NORMALLY

MODEL FIT INFORMATION

Number of Free Parameters 130

Chi-Square Test of Model Fit

Value	308.413*
Degrees of Freedom	189
P-Value	0.0000

* The chi-square value for MLM, MLMV, MLR, ULSMV, WLSM and WLSMV cannot be used for chi-square difference testing in the regular way. MLM, MLR and WLSM chi-square difference testing is described on the Mplus website. MLMV, WLSMV, and ULSMV difference testing is done using the DIFFTEST option.

RMSEA (Root Mean Square Error Of Approximation)

Estimate	0.062	
90 Percent C.I.	0.050	0.075
Probability RMSEA <= .05	0.056	

CFI/TLI

CFI	0.959
TLI	0.950

Chi-Square Test of Model Fit for the Baseline Model

Value	3177.507
Degrees of Freedom	231
P-Value	0.0000

SRMR (Standardized Root Mean Square Residual)

Value 0.066

Optimum Function Value for Weighted Least-Squares Estimator

Value 0.78162231D+00

MODEL RESULTS

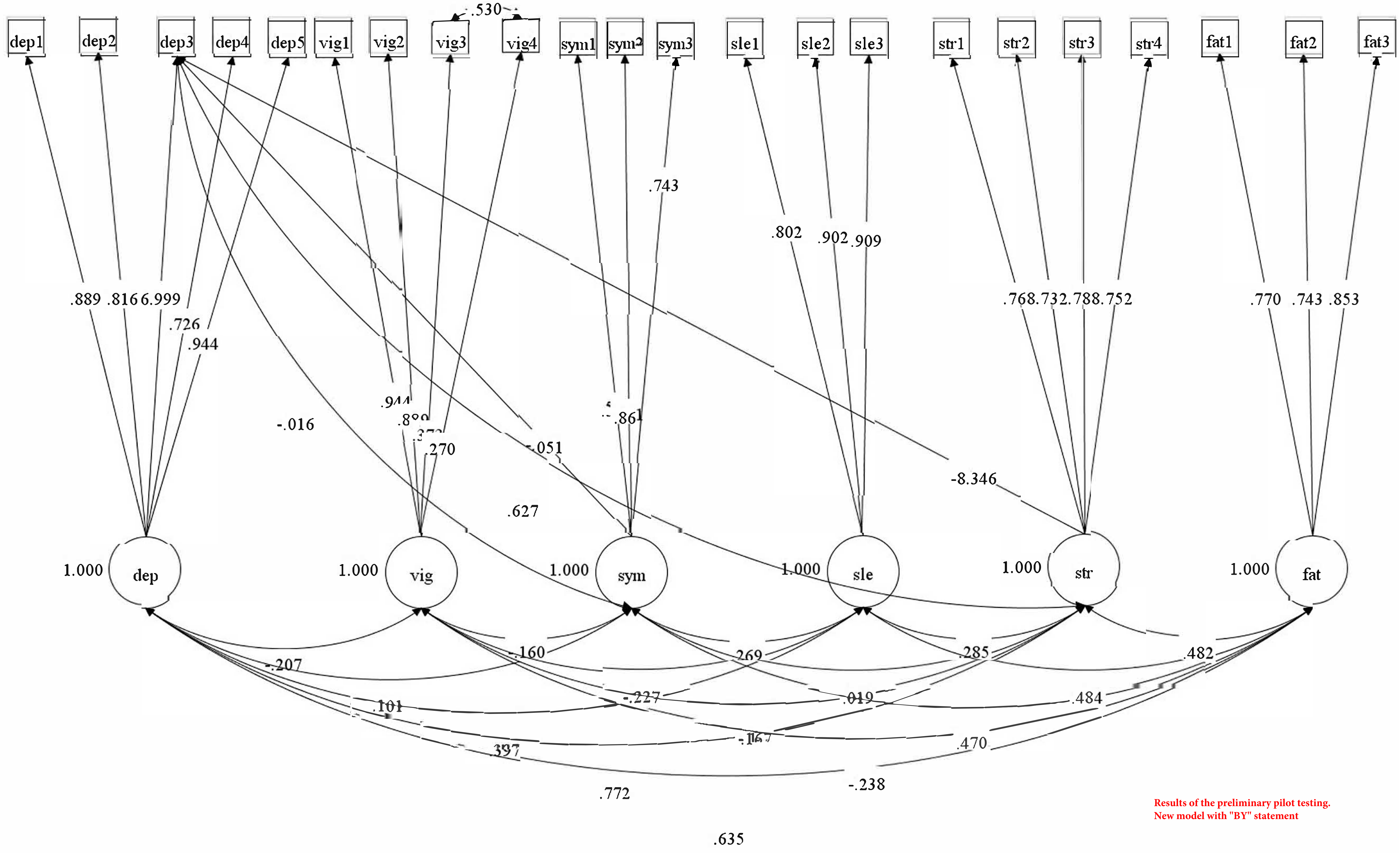

Results of the preliminary pilot testing.
New model with "BY" statement

Mplus Code_Hypothesized CFA

```
TITLE: MTDS 6- factor CFA with MLR;
DATA: File is SPSS to Mplus.dat;
VARIABLE:
NAMES ARE
Gender Age Sport Training
Countie Program Level
dep1 dep2 dep3 dep4 dep5
vig1 vig2 vig3 vig4
sym1 sym2 sym3
sle1 sle2 sle3
str1 str2 str3 str4
fat1 fat2 fat3;

USEVARIABLES are
dep1 dep2 dep3 dep4 dep5
vig1 vig2 vig3 vig4
sym1 sym2 sym3
sle1 sle2 sle3
str1 str2 str3 str4
fat1 fat2 fat3;


ANALYSIS: ESTIMATOR=MLR;


MODEL:   f1 by dep1 dep2 dep3 dep4 dep5; !depression
         f2 by vig1 vig2 vig3 vig4;      !vigour
         f3 by sym1 sym2 sym3;           !physical symptoms
         f4 by sle1 sle2 sle3;           !sleep disturbances
         f5 by str1 str2 str3 str4;      !stress
         f6 by fat1 fat2 fat3;           !fatigue


OUTPUT: TECH1 STDY MOD;

!TECH1 - parameter specification
!STDY - standardized solution
!MOD - modification indices
```

Mplus Code_Alternative CFA

```
TITLE: MTDS 6- factor CFA with MLR;
DATA: File is SPSS to Mplus.dat;
VARIABLE:
NAMES ARE
Gender Age Sport Training
Countie Program Level
dep1 dep2 dep3 dep4 dep5
vig1 vig2 vig3 vig4
sym1 sym2 sym3
sle1 sle2 sle3
str1 str2 str3 str4
fat1 fat2 fat3;

USEVARIABLES are
dep1 dep2 dep3 dep4 dep5
vig1 vig2 vig3 vig4
sym1 sym2 sym3
sle1 sle2 sle3
str1 str2 str3 str4
fat1 fat2 fat3;


ANALYSIS: ESTIMATOR=MLR;


MODEL:   f1 by dep1 dep2 dep3 dep4 dep5; !depression
         f2 by vig1 vig2 vig3 vig4;      !vigour
         f3 by sym1 sym2 sym3;           !physical symptoms
         f4 by sle1 sle2 sle3;           !sleep disturbances
         f5 by str1 str2 str3 str4;      !stress
         f6 by fat1 fat2 fat3;           !fatigue
         str4 WITH str1;
         vig4 WITH vig3;
         fat2 WITH fat1;


OUTPUT: TECH1 STDY MOD;

!TECH1 - parameter specification
!STDY - standardized solution
!MOD - modification indices
```

Mplus Code_MIMIC

```
TITLE: MTDS MIMIC without age and county;
DATA: File is Dataset MIMIC_1.dat;
VARIABLE:
NAMES =
Gender Sport County Age Training Program Level
dep1 dep2 dep3 dep4 dep5
vig1 vig2 vig3 vig4
sym1 sym2 sym3
sle1 sle2 sle3
str1 str2 str3 str4
fat1 fat2 fat3;

MISSING = ALL (-999);

USEVARIABLES =
dep1 dep2 dep3 dep4 dep5
vig1 vig2 vig3 vig4
sym1 sym2 sym3
sle1 sle2 sle3
str1 str2 str3 str4
fat1 fat2 fat3
Gender Sport Training Program Level;

MISSING = ALL (-999);

ANALYSIS: ESTIMATOR=MLR;

MODEL:   f1 by dep1 dep2 dep3 dep4 dep5; !depression
         f2 by vig1 vig2 vig3 vig4;      !vigour
         f3 by sym1 sym2 sym3;           !physical symptoms
         f4 by sle1 sle2 sle3;           !sleep disturbances
         f5 by str1 str2 str3 str4;      !stress
         f6 by fat1 fat2 fat3;           !fatigue
         vig1@0
         f1 on Gender Sport Training Program Level;
         f2 on Gender Sport Training Program Level;
         f3 on Gender Sport Training Program Level;
         f4 on Gender Sport Training Program Level;
         f5 on Gender Sport Training Program Level;
         f6 on Gender Sport Training Program Level;
         str4 with str1; !(133.13- EPC.45)
         vig4 with vig3; !(94.10- EPC.29)
         fat2 with fat1; !(45.33- EPC.30)


OUTPUT: SAMPSTAT TECH4 STAND MOD (ALL);

!TECH4 - parameter specification
!STDY - standardized solution
!MOD - modification indices
```

Mplus Code_Extended MIMIC

```
TITLE: MTDS Extended MIMIC model 2;
DATA: File is Dataset MIMIC_1.dat;
VARIABLE:
NAMES ARE
Gender Sport County Age Training Program Level
dep1 dep2 dep3 dep4 dep5
vig1 vig2 vig3 vig4
sym1 sym2 sym3
sle1 sle2 sle3
str1 str2 str3
str4 fat1 fat2 fat3;

MISSING=ALL (-999);

USEVARIABLES =
Gender Sport Program Level
dep1 dep2 dep3 dep4 dep5
vig1 vig2 vig3 vig4
sym1 sym2 sym3
sle1 sle2 sle3
str1 str2 str3 str4
fat1 fat2 fat3 Load;

DEFINE: Load=0; if Training>10 then Load=1;
!I create a dummy variable Load
!(1- more than 10 hours of training per week
!; 0- less than 10 hours training per week)

ANALYSIS: ESTIMATOR=MLR;


MODEL:   f1 by dep1 dep2 dep3 dep4 dep5; !depression
         f2 by vig1 vig2 vig3 vig4;      !vigour
         f3 by sym1 sym2 sym3;           !physical symptoms
         f4 by sle1 sle2 sle3;           !sleep disturbances
         f5 by str1 str2 str3 str4;      !stress
         f6 by fat1 fat2 fat3;           !fatigue
         f1 on Gender Sport Program Level Load;
         f2 on Gender Sport Program Level Load;
         f3 on Gender Sport Program Level Load;
         f4 on Gender Sport Program Level Load;
         f5 on Gender Sport Program Level Load;
         f6 on Gender Sport Program Level Load;
         str4 WITH str1; !error covariance
         vig4 WITH vig3; !error covariance
         fat2 WITH fat1; !error covariance

dep2 dep3 dep4 dep5 on Gender Sport Program Level Load;
vig2 vig3 vig4      on Gender Sport Program Level Load;
sym2 sym3           on Gender Sport Program Level Load;
sle2 sle3           on Gender Sport Program Level Load;
str2 str3 str4      on Gender Sport Program Level Load;
fat2 fat3           on Gender Sport Program Level Load;
!All indicators, except one, of each latent variable are regressed on
!covariates for the purpose of model identification, the first
!indicators for factors are not regressed on the covariates
!(Kaplan, 2000)

OUTPUT: SAMPSTAT STAND TECH4;
```
